# Spatially Separated Molecular Activation Over Dual Sites in Sn‐Doped ZnGa_2_O_4_/SnO_2_ Photocatalysts Toward Complete VOCs Mineralization

**DOI:** 10.1002/advs.202509891

**Published:** 2025-08-22

**Authors:** Xianli Hu, Bin Liu, Hao Ma, Ruimei Fang, Ting Zhang, Yuhan Li, Behzad Rezaei, Fan Dong

**Affiliations:** ^1^ National Research Base of Intelligent Manufacturing Service College of Environment and Resources Chongqing Technology and Business University Chongqing 400067 China; ^2^ Research Center for Carbon‐Neutral Environmental & Energy Technology Institute of Fundamental and Frontier Sciences University of Electronic Science and Technology of China Chengdu 611731 China; ^3^ Department of Chemistry Isfahan University of Technology Isfahan 84156–83111 Iran

**Keywords:** electronic transfer, photocatalysis, spatially separated activation, toluene degradation

## Abstract

Photocatalysis holds great promise for air pollutant remediation; however, its efficiency is often limited by charge recombination and humidity interference. Herein, Sn‐doped ZnGa_2_O_4_ loaded SnO_2_ nanoparticles (SZGO) is in situ constructed via a one‐step hydrothermal method. The doped Sn atoms act as electron‐trapping centers and form interfacial Sn─O channels between ZnGa_2_O_4_ and SnO_2_, enabling directional electron migration. Hydrophilic ZnGa_2_O_4_ domains selectively adsorb H_2_O, generating hydroxyl radicals (·OH) that facilitate aromatic ring cleavage, while electron‐rich SnO_2_ domains activate O_2_ to produce superoxide (·O_2_^−^) and singlet oxygen (^1^O_2_), leading to complete degradation of carbonaceous intermediates. The optimized SZGO achieves nearly 100% toluene degradation and 99.2% mineralization efficiency, with excellent stability across a wide humidity range (0–100%) and over 12 continuous cycles (720 min). Additionally, SZGO demonstrates broad‐spectrum reactivity toward other volatile organic compounds (VOCs), such as acetone and formaldehyde. This work offers a versatile design strategy that couples directional charge transport with site‐specific molecular activation, providing new insights for the development of highly stable photocatalysts for environmental purification.

## Graphical abstract

The graphical abstract illustrates the photocatalytic mechanism of Sn‐doped ZnGa_2_O_4_ with in situ loaded SnO_2_ (SZGO), highlighting directional electron migration and selective adsorption/activation. ZnGa_2_O_4_ domains facilitate H_2_O activation to generate ·OH radicals, while SnO_2_ domains enriched with electrons promote O_2_ activation to form ·O_2_^−^ and ^1^O_2_, enabling efficient mineralization of VOCs (e.g., toluene) into CO_2_ and H_2_O under UV irradiation.

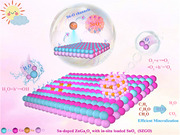

## 1 Introduction

Toluene, a representative VOC within the benzene series, is extensively utilized as a solvent and key intermediate in various industries. Its high volatility contributes to significant atmospheric pollution through incomplete combustion and evaporation.^[^ ^1^, ^2^, ^3^ ^]^ However, the degradation and complete mineralization of toluene are hindered by its stable π‐conjugated aromatic structure, which poses challenges for benzene‐ring cleavage, a critical step in pollutant mineralization.^[^ ^4^, ^5^, ^6^ ^]^ Photocatalysis has emerged as a promising and sustainable approach owing to mild reaction conditions, low energy consumption, and potential for complete mineralization.^[^ ^7^, ^8^ ^]^ Nevertheless, practical applications of photocatalysis in toluene degradation are still limited by relatively low efficiency and sensitivity to environmental humidity.^[^ ^9^ ^]^ Thus, developing advanced photocatalysts capable of overcoming these limitations and enabling effective, durable mineralization of toluene is urgently needed.

To achieve complete photocatalytic mineralization of toluene, photocatalysts with strongly oxidative valence bands and positive potentials are ideal.^[^ ^10^, ^11^ ^]^ Among them, ZnGa_2_O_4_ stands out due to its favorable electronic structure.^[^ ^12^, ^13^ ^]^ ZnGa_2_O_4_ is notable for its wide bandgap, highly positive valence band, and robust Zn─Ga orbital hybridization, which confer exceptional oxidative capacity and thermal stability.^[^ ^14^, ^15^ ^]^ These features also confer intrinsic potential for efficient charge separation, critical for deep mineralization of aromatic pollutants like toluene.^[^ ^16^, ^17^ ^]^ Despite these advantages, pristine ZnGa_2_O_4_ is limited by poor solar utilization and high photogenerated charge carrier recombination, reducing its photocatalytic efficiency.^[^ ^18^ ^]^ To overcome these limitations, various modification strategies—such as metal‐ion doping and heterojunction construction—have been explored to enhance light absorption and charge carrier dynamics.^[^ ^19^, ^20^ ^]^ Metal‐ion doping, valued for its simplicity, modulates electronic structures, promotes electron localization, and forms interfacial electric fields or Schottky barriers to suppress recombination.^[^ ^21^, ^22^ ^]^ Yet, excessive electron accumulation at dopant sites can trigger trap‐mediated recombination, lowering efficiency.^[^ ^23^ ^]^ Therefore, designing directional electron transport pathways within ZnGa_2_O_4_ is critical to minimize recombination and maximize photocatalytic performance. Furthermore, the application of ZnGa_2_O_4_ in gaseous VOC degradation, particularly toluene, is hindered by environmental factors, notably atmospheric humidity.^[^ ^24^ ^]^ Excessive ambient H_2_O molecules compete for active sites, impeding pollutants and O_2_ adsorption and reducing photocatalytic efficiency and stability.^[^ ^25^ ^]^ Current strategies addressing humidity interference mainly involve introducing hydrophobic components to reduce H_2_O adsorption^[^ ^26^, ^27^ ^]^; however, these approaches inadvertently impair the generation of hydroxyl radicals (·OH), crucial for toluene's aromatic ring cleavage.^[^ ^28^ ^]^ Therefore, approaches that balance reduced H_2_O interference with sustained reactive oxygen species (ROS) production are urgently required to ensure sustained catalytic activity and deep pollutant oxidation.

Given that pristine ZnGa_2_O_4_ typically exhibits inherent surface hydrophilicity due to abundant hydroxyl groups, a rational design combining ZnGa_2_O_4_ with complementary co‐catalysts capable of selective molecular adsorption could effectively resolve these issues. Among potential candidates, SnO_2_ stands out due to its robust O_2_ adsorption properties, high electron affinity, and excellent electron‐transfer capabilities.^[^ ^29^, ^30^ ^]^ Constructing ZnGa_2_O_4_/SnO_2_ heterojunctions could strategically achieve spatially separated adsorption‐activation sites: the hydrophilic ZnGa_2_O_4_ domains preferentially adsorb H_2_O molecules, promoting efficient generation of ·OH radicals for benzene‐ring opening, whereas SnO_2_ domains enriched with electron density favor selective O_2_ adsorption, facilitating the production of ·O_2_^−^ and ^1^O_2_ radicals essential for oxidizing carbonaceous intermediates.^[^ ^31^ ^]^ This spatial separation of reactant molecules (H_2_O and O_2_) minimizes site competition and ensures simultaneous, complementary ROS generation with high selectivity and accessibility. In contrast, conventional single‐phase catalysts often suffer from competitive adsorption or unbalanced ROS formation, limiting full mineralization.^[^ ^5^, ^32^ ^]^ Strategic metal‐ion doping may further narrow ZnGa_2_O_4_’s bandgap, enhancing light response and directional electron transfer at the heterointerface. However, systematic studies on dual‐site activation systems using metal‐doped ZnGa_2_O_4_/SnO_2_ heterostructures for humidity‐tolerant, high‐efficiency VOC photocatalysis remain limited.

Motivated by these unresolved challenges, this study develops a novel photocatalytic system by integrating Sn‐doped ZnGa_2_O_4_ with in situ loaded SnO_2_ co‐catalysts. The optimized SZGO photocatalyst achieves exceptional photocatalytic activity (almost 100%), mineralization efficiency (99.2%), stability (720 min, 12 cycles), and humidity adaptability (0–100%) in toluene oxidation. Advanced characterization techniques and DFT calculations elucidate the mechanisms driving this performance: i) Sn–O bonds at the heterojunction interface form efficient electron‐transfer channels, enabling directional electron migration and high carrier utilization; ii) the heterojunction's distinct components facilitate spatially separated adsorption and activation of H_2_O and O_2_, minimizing site competition and enhancing multiple ROS generation; iii) ·OH radicals drive aromatic ring cleavage, while ·O_2_^−^ and ^1^O_2_ oxidize carbonaceous intermediates, synergistically mineralizing toluene into CO_2_ and H_2_O. Unlike conventional strategies that rely on hydrophobic modifications or single‐site activation, which often compromise ROS generation or stability, our dual‐site approach leverages Sn–O channels and polarity‐opposed domains to achieve synergistic H_2_O and O_2_ activation, ensuring robust performance across diverse environmental conditions.

## 2 Results and Discussion

### 2.1 Synthesis and Structural Characterizations

A series of Sn doped ZnGa_2_O_4_ photocatalysts with in situ loaded SnO_2_ (denoted as SZGO‐x, where x indicates the relative amount of Sn precursor) were synthesized via a one‐step hydrothermal method (160 °C, 24 h), as detailed in Figure S1 (Supporting Information). Among these, SZGO‐3 (hereafter SZGO) exhibited the highest photocatalytic activity and was selected as the representative sample. For comparison, reference samples including pristine ZnGa_2_O_4_ (ZGO), Sn‐doped ZnGa_2_O_4_ without SnO_2_ (SnZGO), and SnO_2_‐loaded ZnGa_2_O_4_ (SnO_2_/ZGO), were prepared. Detailed synthetic procedures and reagent dosages are provided in Table S1 (Supporting Information).

**Figure 1:**
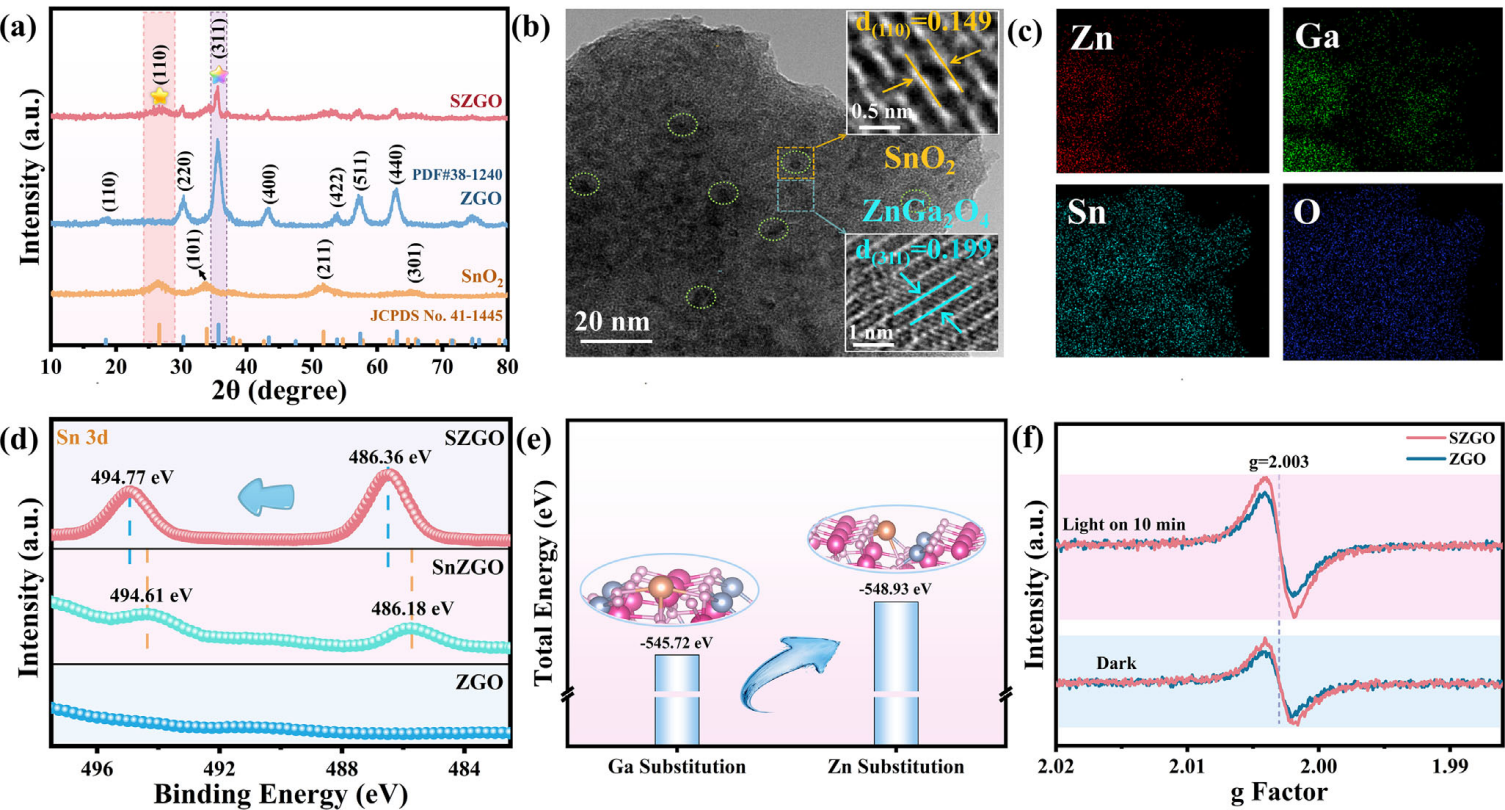
a) XRD pattern of the as‐prepared samples. b) TEM image and the corresponding HRTEM images of SZGO, and c) the corresponding EDS mapping patterns of Zn, Ga, Sn, and O. d) Sn 3d XPS spectra of ZGO, SnZGO, and SZGO. e) Calculated total energy for different substitution models. f) EPR spectra of ZGO and SZGO.

X‐ray diffraction (XRD) analysis confirmed the crystal structure of SZGO. All composite samples display characteristic diffraction peaks at 35.62°, 57.2° and 62.73°, corresponding to the (311), (511) and (440) crystal planes of ZnGa_2_O_4_ (ZGO, JCPDS No. 38–1240),^[^ ^33^ ^]^ indicating retention of the pristine crystal structure after Sn incorporation (**Figure** **1**a; Figure S2, Supporting Information). Notably, with increasing Sn content, a broad peak at 26.5° emerges, assigned to the (110) plane of tetragonal SnO_2_ (JCPDS No. 41–1445),^[^ ^34^ ^]^ confirming in situ SnO_2_ formation. Meanwhile, a slight shift of ZnGa_2_O_4_ peaks to lower angles (Δ2θ ≈ 0.1°) suggests lattice expansion due to Sn^4+^ doping, consistent with the larger ionic radius of Sn^4+^ (0.69 Å) compared to Zn^2+^ (0.60 Å). The XRD patterns of SnZGO (Sn‐doped only) and SnO_2_/ZGO (SnO_2_‐loaded only) are presented in Supporting Information (Figure S3, Supporting Information). Compared to ZGO, SnZGO only exhibited angular shifts, while SnO_2_/ZGO only additionally detected characteristic peaks belonging to SnO_2_.

To further elucidate the microscopic morphology and interface structure of the composites, scanning electron microscope (SEM), transmission electron microscopy (TEM) and high‐resolution TEM (HRTEM) analyses were performed (Figure 1b; Figure S4a–d, Supporting Information). Pristine ZGO exhibits irregular spherical particles with lattice fringes of 0.2 nm, corresponding to the (311) plane. In SZGO, SnO_2_ nanoparticles (2‐3 nm) are uniformly anchored on ZnGa_2_O_4_, showing lattice fringes of 0.15 nm for the SnO_2_ (110) plane. The coherent interface between ZnGa_2_O_4_ and SnO_2_ indicates a tight heterojunction, facilitating charge transfer. Elemental mapping from energy‐dispersive X‐ray spectroscopy (EDS, Figure 1c) further confirms the homogeneous distribution of Zn, Ga, O, and Sn, validating the simultaneous doping and surface deposition of Sn species. X‐ray photoelectron spectroscopy (XPS) further verifies Sn^4+^ incorporation (Figure 1d). Compared to SnZGO, the further shift of Sn 3d peaks in SZGO supports the presence of SnO_2_, consistent with HRTEM and XRD evidence.

Nitrogen adsorption‐desorption isotherms (Figure S5a–d, Supporting Information) reveal type IV mesoporous structures for both ZGO and SZGO. SZGO exhibits a 1.23‐fold higher specific surface area (Table S2, Supporting Information), attributed to SnO_2_‐induced particle size reduction, enhancing active site availability,^[^ ^35^ ^]^ a conclusion also supported by SEM observations of improved particle uniformity in SZGO (Figure S4a,c, Supporting Information). To rationalize the doping mechanism observed experimentally, DFT calculations indicate that Sn^4+^ preferentially substitutes Zn^2+^ over Ga^3+^ due to lower formation energy (Figure 1e). This aliovalent substitution usually induces oxygen vacancies to restore local electroneutrality,^[^ ^36^ ^]^ which can be confirmed by the electron paramagnetic resonance (EPR) spectra. EPR spectra at 77 K under vacuum show intensified oxygen vacancy signals in SZGO (Figure 1f), suggesting defect‐mediated charge separation and molecular activation. These results collectively confirm the successful synthesis of Sn‐doped ZnGa_2_O_4_/SnO_2_ heterostructures with optimized structural and electronic properties.

### 2.2 Photocatalytic Performance and Mechanism

**Figure 2:**
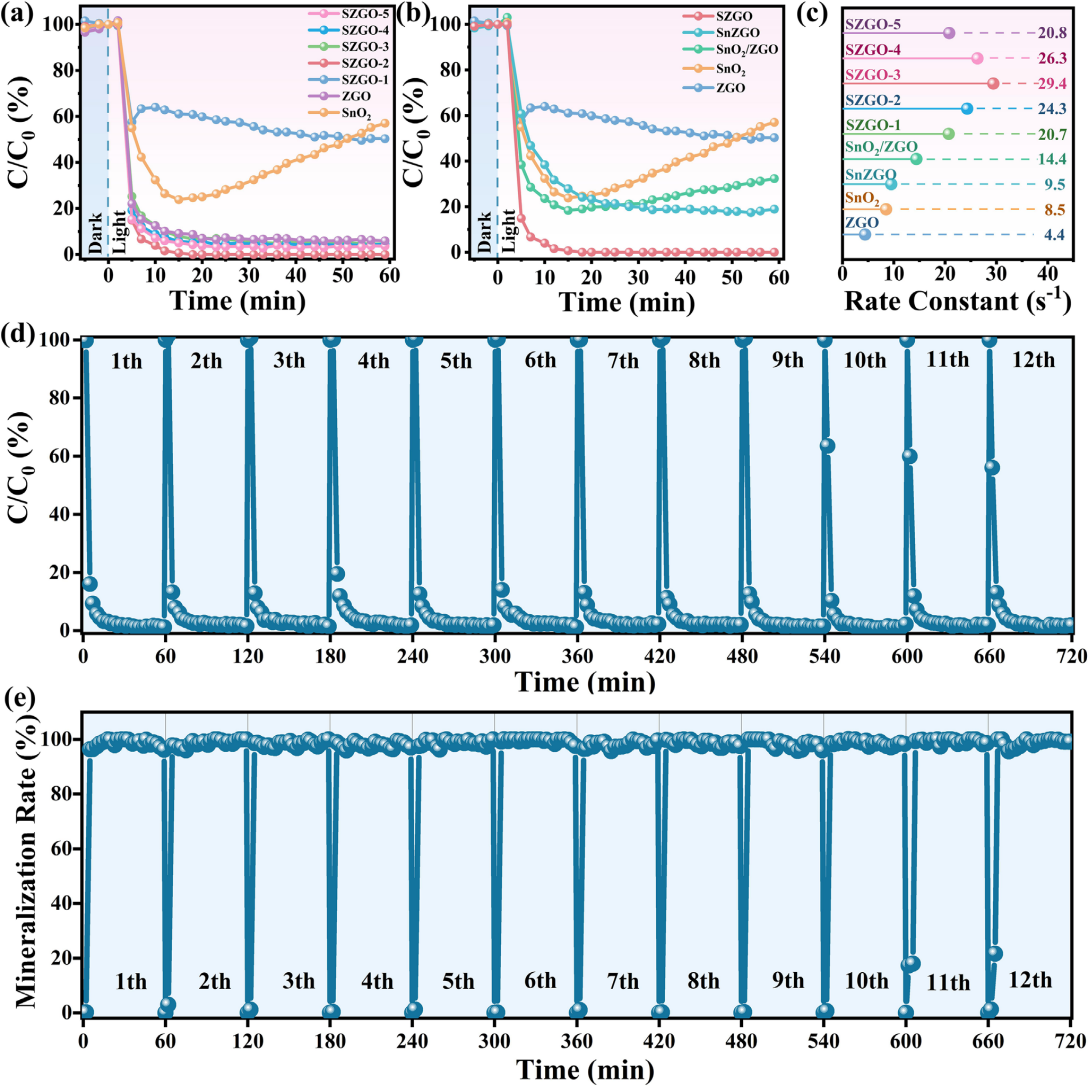
a, b) The photocatalytic toluene degradation efficiency of the as‐prepared samples, and c) the corresponding first‐order rate constant. d) Photocatalytic toluene degradation cycle test of SZGO, and e) the corresponding mineralization rate.

The photocatalytic performance of the as‐prepared samples was evaluated for toluene mineralization in a continuous flow system under UV irradiation (light source: 150 W, λ < 365 nm, initial toluene concentration: 60 ppm, total flow rate: 1 L·min^−1^, relative humidity: 50%). Compared to pristine ZnGa_2_O_4_ (ZGO), Sn‐doped ZnGa_2_O_4_ with in situ loaded SnO_2_ (SZGO‐x) exhibits markedly enhanced activity, displaying a volcano‐type trend with increasing Sn loading (**Figure** **2**a). This trend arises from a synergy between optimized charge separation and molecular activation at moderate Sn doping. At moderate doping levels, Sn^4+^ ions substitute Zn^2+^ in the ZnGa_2_O_4_ lattice, creating shallow electron traps that effectively capture photogenerated electrons and facilitate their directional migration. However, at excessive doping levels, the density of Sn dopants becomes high enough that additional Sn^4+^ centers act as deep‐level trap states, which instead facilitate non‐radiative recombination pathways.^[^ ^37^, ^38^ ^]^ Among all the samples, SZGO‐3 (hereafter SZGO) achieves almost 100% toluene degradation and an apparent mineralization efficiency of 99.2% (Figure 2a; Figure S6a, Supporting Information), surpassing commercial TiO_2_ (Figure S6b, Supporting Information). The observed mineralization efficiency occasionally exceeding 100% is attributed to the additional oxidation of pre‐adsorbed carbonaceous contaminants accumulated on the catalyst surface during the initial dark adsorption stage, as confirmed by total organic carbon analysis.^[^ ^39^ ^]^ To elucidate the roles of Sn doping and SnO_2_ loading, SnZGO (Sn‐doped only) and SnO_2_/ZGO (SnO_2_‐loaded only) were tested (Figure 2b). SZGO outperforms both, with the loading of SnO_2_ further enhancing the catalytic activity of SnZGO, indicating a synergistic effect driven by the electron‐trapping of doped Sn atoms and SnO_2_‐mediated charge transfer. Kinetic analysis revealed a first‐order rate constant of 29.4 s^−1^ for SZGO, 6.8‐fold higher than ZGO's 4.4 s^−1^ (Figure 2c). SZGO also demonstrates excellent stability, maintaining high CO_2_ selectivity over 12 consecutive cycles (720 min) (Figure 2d,e). To further evaluate the durability of SZGO under long‐term operation, post‐reaction characterizations were performed after 12 consecutive photocatalytic cycles. As shown in Figure S7 (Supporting Information), the XRD pattern of the used catalyst remains virtually unchanged, confirming phase stability. FTIR spectra reveal no significant loss of characteristic surface functional groups, and XPS analysis shows negligible shifts in Zn 2p, Sn 3d and Ga 2p peaks, indicating the preservation of surface chemical states. These results collectively demonstrate that SZGO retains both its structural integrity and surface reactivity, validating its robustness for practical photocatalytic applications.

**Figure 3:**
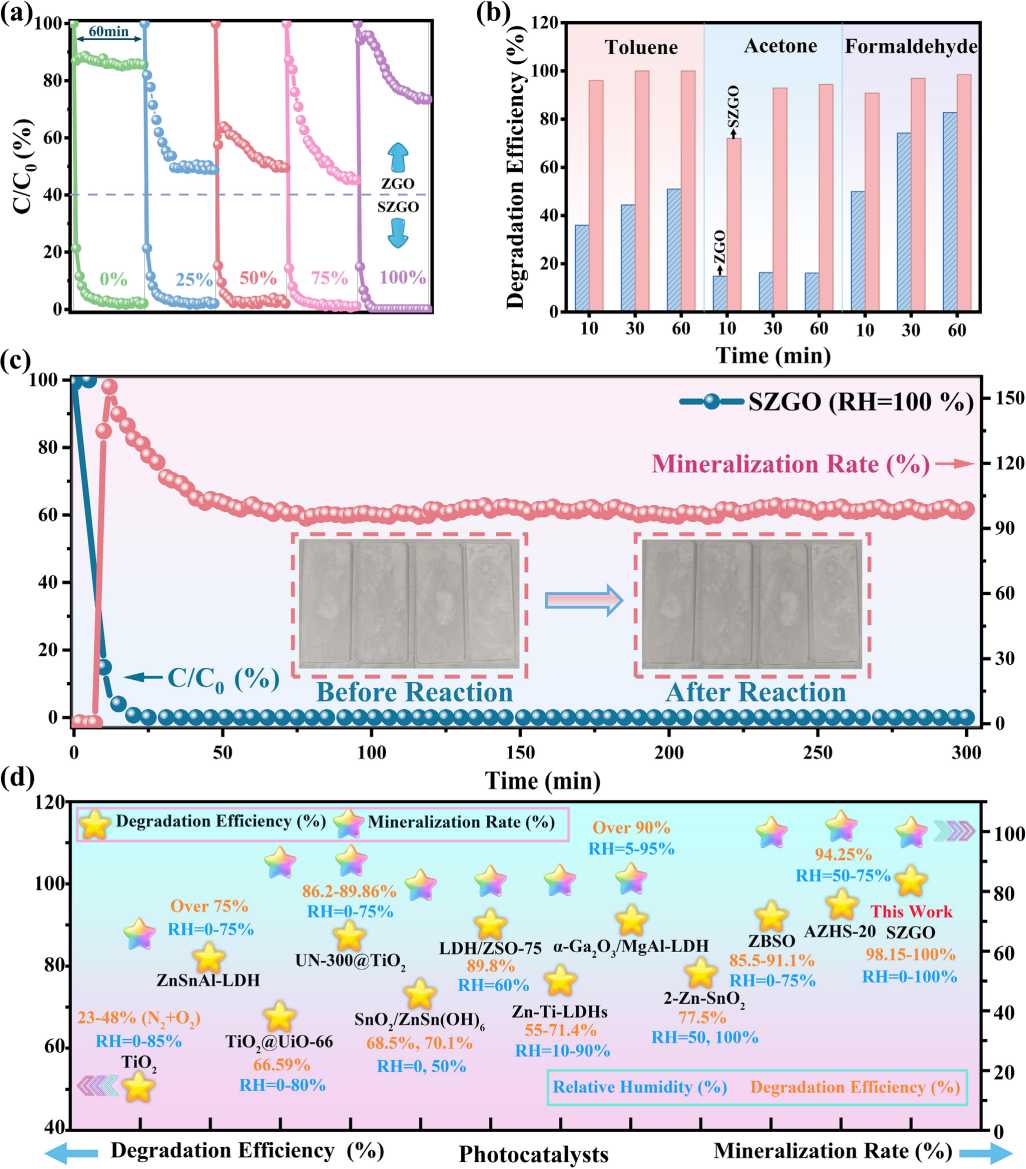
The photocatalytic degradation efficiency under different a) relative humidity conditions and b) volatile organic compounds. c) Long‐term photocatalytic toluene degradation at 100% RH over 300‐min test, with inset images showing the samples before and after the cyclic test. d) Comparative Photocatalytic Degradation Efficiency of Representative Photocatalysts.

Controlled humidity experiments were conducted to evaluate photocatalytic performance under varying relative humidity (RH) (**Figure** **3**a). ZGO exhibited a marked decline in activity under both extremely dry (0% relative humidity, RH) and highly humid (100% RH) conditions, reflecting its poor environmental adaptability. In contrast, the SZGO sample maintained excellent degradation efficiency across the full humidity range, achieving 98.15% degradation at 0% RH and 100% at 100% RH. Notably, its performance remained stable under 100% RH over a 5‐h continuous test (Figure 3c), and no carbonaceous deposits were observed on the catalyst surface (insets of Figure 3c), indicating strong resistance to intermediate accumulation.^[^ ^40^ ^]^ The remarkable humidity adaptability arises from a dual‐site activation mechanism facilitated by the site‐specific heterojunction structure. Moreover, the broad‐spectrum applicability of SZGO was further evaluated using acetone and formaldehyde as representative pollutants (Figure 3b). For acetone (400 ppm), SZGO achieved 94.5% degradation and 96.2% mineralization efficiency, representing over sixfold improvements compared to ZGO (Figure S8a, Supporting Information). SZGO also exhibited excellent formaldehyde degradation performance (Figure S8b, Supporting Information), which confirmed that this dual‐site strategy is versatile and can be extended to other photocatalytic systems. As shown in Figure 3d and summarized in Table S3 (Supporting Information), compared to state‐of‐the‐art photocatalysts, such as TiO_2_‐based composites or LDH, SZGO achieves superior toluene mineralization and unmatched humidity tolerance (0–100%). These advantages are attributed to the synergistic integration of Sn–O interfacial electron‐transfer channels, a dual‐site activation mechanism, and spatially coordinated ROS generation.

**Figure 4:**
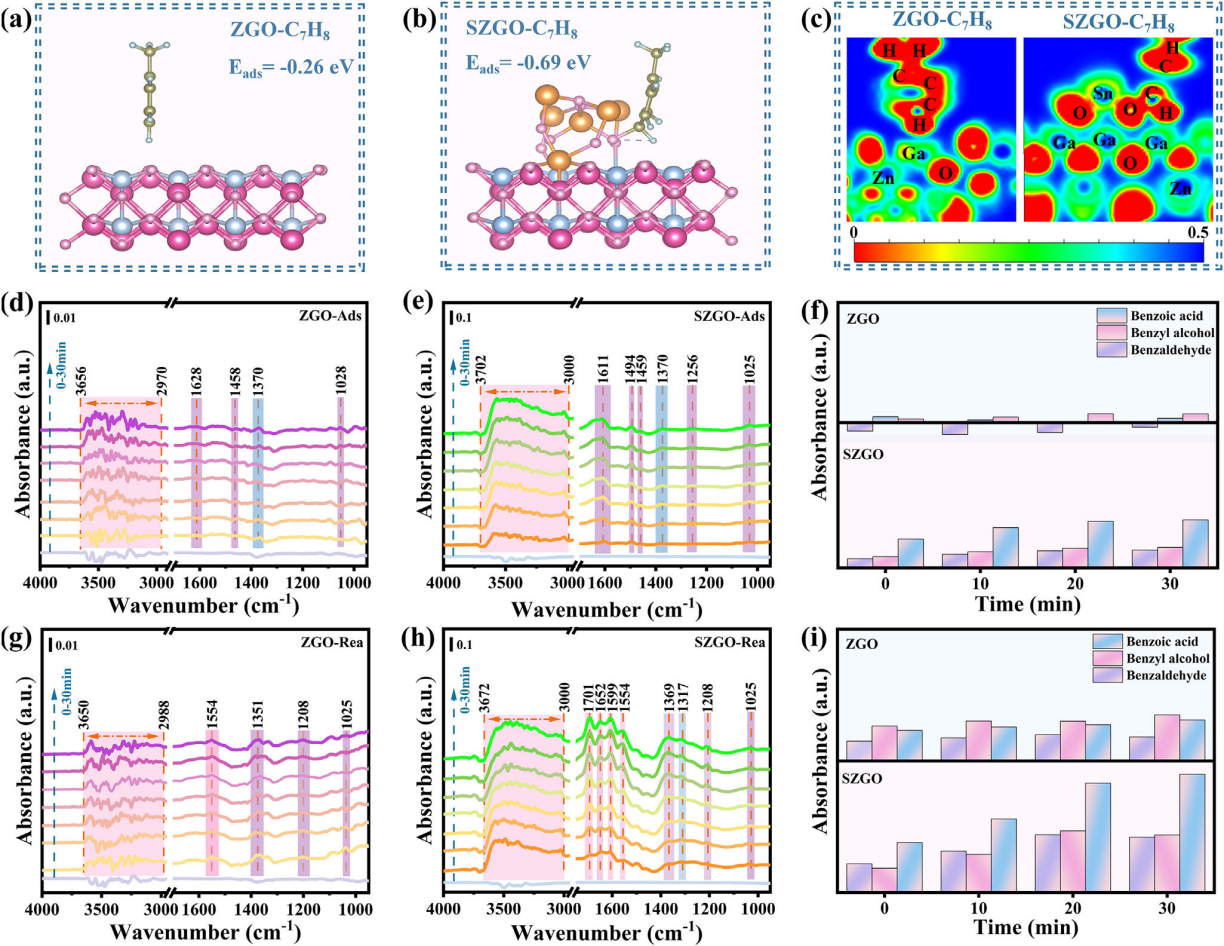
The adsorption configuration of toluene on a) ZGO and b) SZGO, and c) the corresponding electron local functions (ELF). In situ DRIFTS spectra of toluene d, e) adsorption and and g, h) photo‐oxidation on the ZGO and SZGO. Species evolution during the toluene f) adsorption and i) photocatalytic degradation processes.

Subsequently, the interaction between the pollutant toluene and the catalyst was further studied. DFT calculations reveal stronger toluene adsorption on SZGO (E_ads_ = ‐0.69 eV) than ZGO (E_ads_ = ‐0.26 eV) (**Figure** **4**a,b). On the SZGO surface, the toluene molecule adsorbs primarily via π–π interaction between the benzene ring and surface O atoms of SnO_2_ at the interface. Moreover, electron localization function (ELF) analysis indicating C‐H bond elongation on SZGO (Figure 4c), facilitating benzene ring activation. The activation of benzene ring serves as a preconditioning effect: it weakens the electronic structure of the aromatic ring, facilitating subsequent oxidation and ring‐opening steps once the methyl group has been activated. To experimentally verify the adsorption behavior and identify surface intermediates, in situ diffuse reflectance infrared Fourier transform spectroscopy (DRIFTS) was performed under dark conditions to simulate the initial adsorption stage of photocatalysis (Figure 4d,e). The corresponding surface species are summarized in Table S4 (Supporting Information). Upon introducing toluene into the dark reactor, characteristic bands at 1494 and 1459 cm^−1^ (benzene ring skeletal vibrations) and 1611 cm^−1^ (C═C stretching) were detected, confirming molecular adsorption without ring‐opening under light‐free conditions. Additional weak bands at 1370, 1256, and 1025 cm^−1^, attributed to C─O stretching vibrations of benzyl alcohol, indicating limited spontaneous oxidation of toluene. Notably, these signals were more intense on SZGO than on ZGO (Figure 4f), suggesting higher adsorption capacity, consistent with DFT predictions.

Upon UV illumination, the gradual decrease in benzene ring‐associated peaks indicates the consumption and potential ring opening of toluene. For ZGO, bands at 1351, 1208, and 1025 cm^−1^ are observed (Figure 4g), corresponding to the C─O stretching vibrations of benzyl alcohol. However, excessive accumulation of benzyl alcohol may block active sites and hinder further oxidation.^[^ ^41^ ^]^ In contrast, SZGO displays prominent peaks at 1701, 1652, 1599, and 1554 cm^−1^ (Figure 4h,i), assigned to benzoic acid, a crucial intermediate promoting rapid ring‐opening and subsequent mineralization.^[^ ^42^, ^43^ ^]^ Additional minor peaks at 1369, 1208, and 1025 cm^−1^ (benzyl alcohol) and 1317 cm^−1^ (benzaldehyde, C═O stretch) indicate the presence of transient intermediates. This clearly shows that initial oxidation occurs at the methyl group, consistent with widely reported mechanisms of toluene photocatalysis.^[^ ^39^, ^44^ ^]^ Based on these observations, the proposed toluene degradation pathway proceeds as follows: toluene → toluene adsorption (pre‐activation) → benzyl alcohol → benzaldehyde → benzoic acid → ring‐opening → complete mineralization to CO_2_ and H_2_O.

### 2.3 Electron Transfer and Molecular Activation

**Figure 5:**
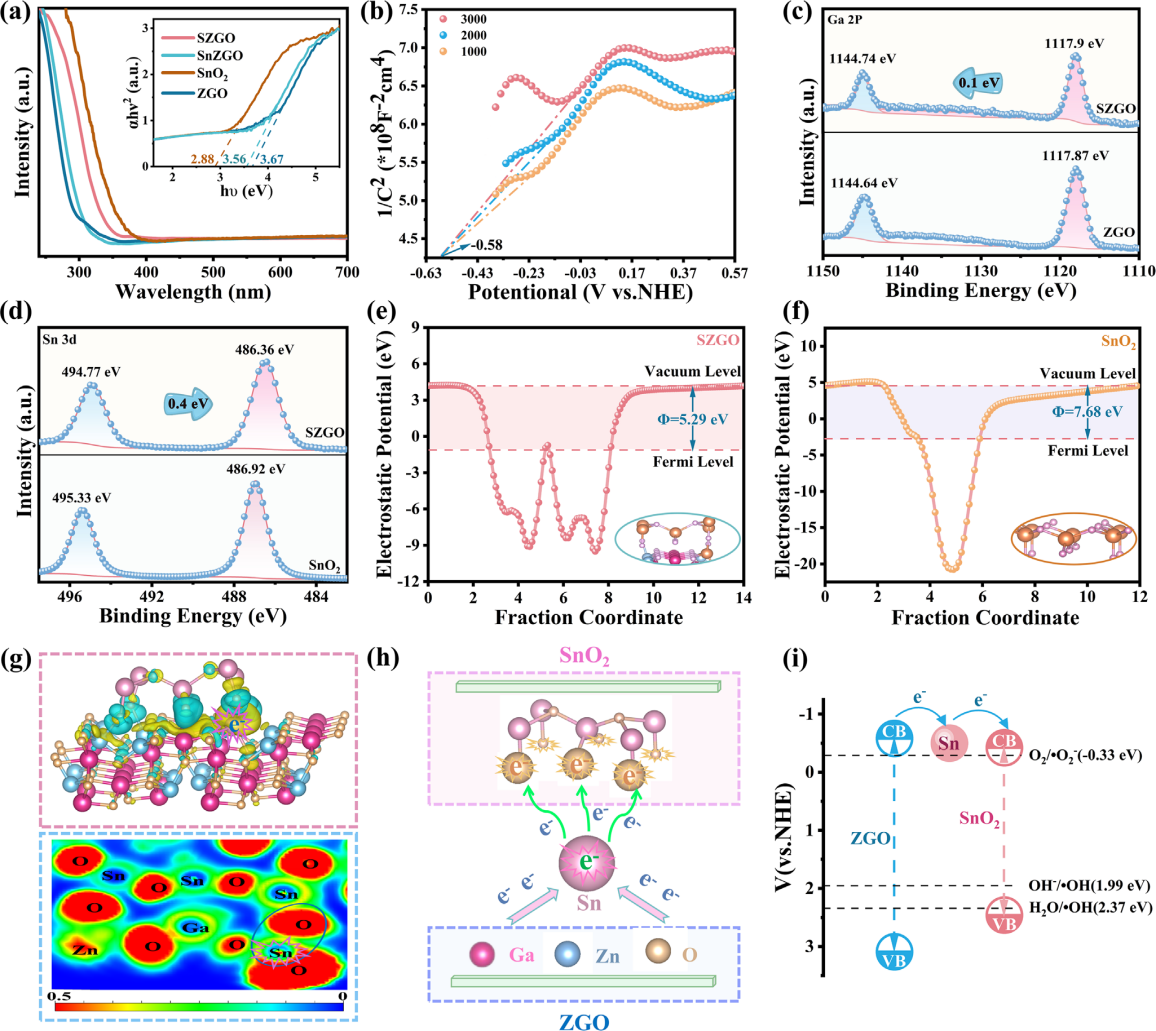
a) UV–vis DRS (illustrated as a graph of hν and (αhν)^2^). b) Mott Schottky spectra of SnZGO. c) Ga 2p XPS spectra of ZGO and SZGO. d) Sn 3d XPS spectra of SnO_2_ and SZGO. Work functions and the relative Fermi levels of e) SZGO and f) SnO_2_. g) Differential charge density and ELF simulation of SZGO, the yellow or blue color of the electron clouds mean that which loses electrons or gains electrons. h) Electron migration pathway diagram, and i) energy band structure diagram of SZGO.

Efficient charge transfer and molecular activation underpin superior photocatalytic performance. To elucidate these processes, we systematically investigated the electronic structure using UV–vis diffuse reflectance spectroscopy (UV–vis DRS), Mott–Schottky analysis, and XPS. As shown in **Figure** **5**a, SZGO exhibits an absorption edge similar to that of pristine ZnGa_2_O_4_ (∼350 nm) but with enhanced UV‐region absorption intensity. Tauc plots indicate a bandgap (E_g_) narrowing from 3.67 eV (ZGO) to 3.56 eV (SnZGO), attributed to Sn doping‐induced intermediate states and defect states within the band structure.^[^ ^45^, ^46^ ^]^ Mott–Schottky measurements (Figure 5b; Figure S9, Supporting Information) reveal conduction band (CB) potentials of ‐0.58 eV (SnZGO) and ‐0.42 eV (SnO_2_), both more negative than the O_2_/·O_2_^–^ reduction potential (−0.33 eV), enabling superoxide radical (·O_2_^–^) generation. Valence band (VB) potentials, calculated via E_g_ = E_VB_ – E_CB_,^[^ ^47^ ^]^ are 2.98 eV (SnZGO) and 2.46 eV (SnO_2_), surpassing the threshold for ·OH formation (2.37 eV), confirming the potential for efficient generation of various ROS.

Building on electronic structure insights, XPS and work function analysis were performed to elucidate the charge transfer dynamics between heterojunction interfaces. As shown in Figure 5c, the Ga 2p spectra of SZGO exhibit peaks at 1144.74 eV (Ga 2p_1/2_) and 1117.9 eV (Ga 2p_3/2_), corresponding to Ga^3+^,^[^ ^48^ ^]^ with a +0.1 eV shift relative to pristine ZGO. Similarly, the Zn 2p binding energies in SZGO also shift toward higher energies (Figure S10, Supporting Information), further confirming modification of the electronic structure. In contrast, Sn 3d peaks in SZGO at 486.4 and 494.8 eV exhibit a negative shift (−0.4 eV) compared to SnO_2_, indicating increased electron density around Sn atoms due to interfacial charge transfer from ZnGa_2_O_4_ to SnO_2_ (Figure 5d). Three peaks at 530.11, 531.68, and 532.68 eV in the O 1s XPS spectra of ZGO (Figure S11, Supporting Information) are assigned to lattice oxygen, adsorbed hydroxyl groups, and chemisorbed H_2_O, respectively.^[^ ^49^, ^50^ ^]^ Notably, SZGO exhibits increased peak areas and ratios for adsorbed hydroxyl groups and H_2_O compared to ZGO, suggesting enhanced surface hydroxylation and H_2_O affinity. The work functions of ZGO, SZGO, and SnO_2_ were calculated as 5.34, 5.29, and 7.68 eV, respectively (Figure 5e,f; Figure S12, Supporting Information). This substantial difference (Δφ ≈ 2.39 eV) generates a built‐in electric field at the heterojunction, forming a Schottky barrier that causes downward band bending.^[^ ^51^ ^]^ Given that electrons spontaneously migrate from regions of lower work function to higher work function, the energy alignment thermodynamically drives photogenerated electrons from ZnGa_2_O_4_ to SnO_2_, while retaining holes in ZnGa_2_O_4_, thereby facilitating efficient charge separation. In addition, Sn incorporation slightly lowers the work function (5.29 eV for SZGO vs 5.34 eV for ZGO) and elevates the Fermi level, further enhancing the interfacial electric field and facilitating directional electron transfer across the heterojunction, thereby promoting efficient charge separation.

DFT calculations, including charge density difference and electron localization function (ELF) analyses, confirmed the critical role of Sn^4+^ doping in SZGO. Charge density difference analysis reveals electron accumulation around Sn^4+^ ions within the ZnGa_2_O_4_ lattice, establishing these ions as electron‐trapping centers due to their high electronegativity (Figure 5g).^[^ ^52^ ^]^ ELF analysis (inset in Figure 5g) further demonstrates strong interactions between oxygen atoms in SnO_2_ and doped Sn^4+^ atoms, forming Sn–O electron‐transfer channels that significantly enhance charge transfer efficiency.^[^ ^53^, ^54^ ^]^ Consequently, Sn^4+^ ions in the ZnGa_2_O_4_ lattice capture electrons and efficiently transfer them to SnO_2_ via these Sn–O channels, promoting spatial separation of electrons (in SnO_2_) and holes (in ZnGa_2_O_4_), thereby minimizing recombination. Schematic diagrams of electron migration pathways and energy band structures are provided in Figure 5h,i, offering comprehensive insights into SZGO's charge transfer dynamics.

**Figure 6:**
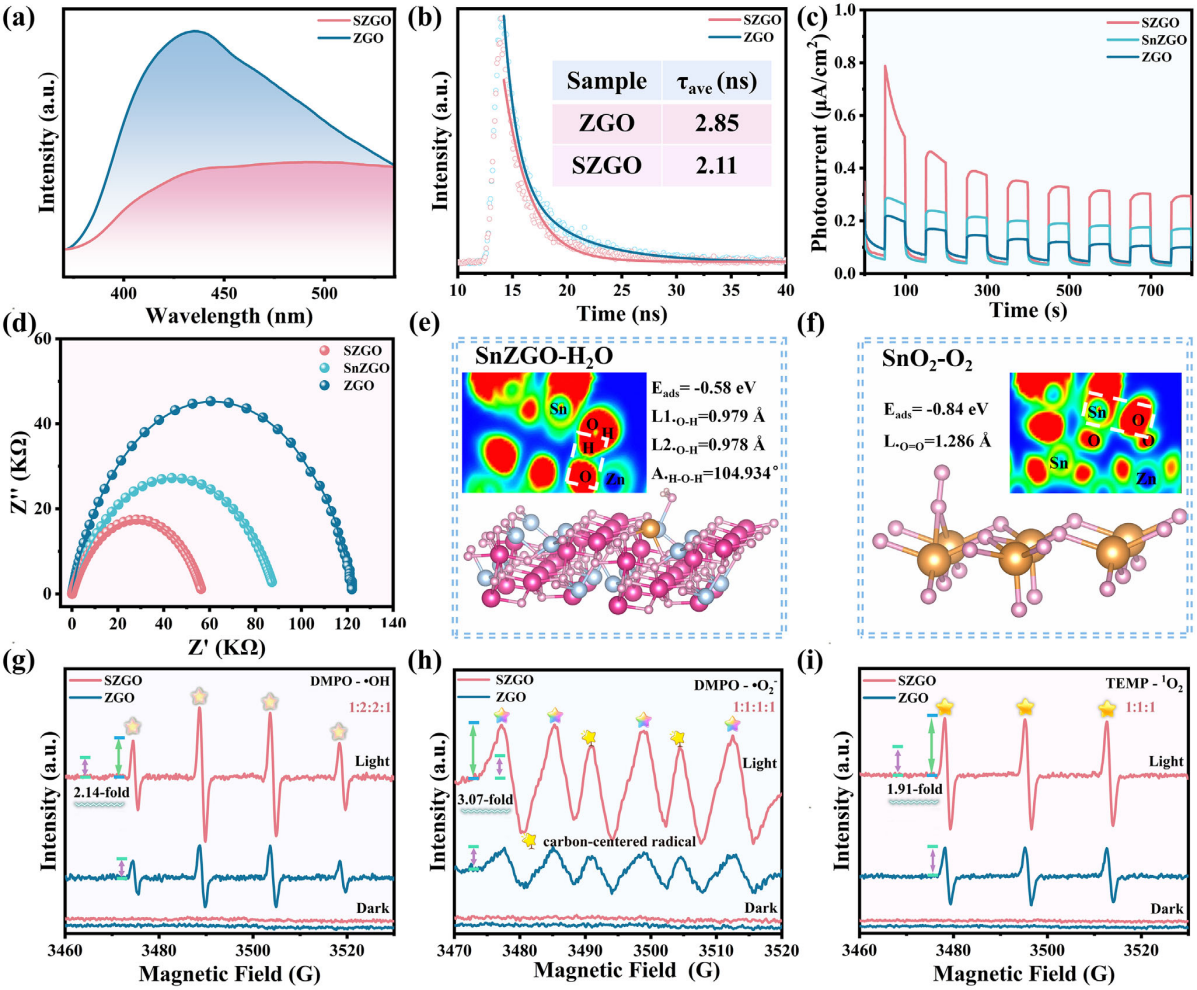
a) PL and b) TRPL decay spectra of ZGO and SZGO. c) Photocurrent responses and d) EIS Nyquist plots measurements of ZGO, SnZGO, and SZGO. The adsorption models of e) H_2_O molecule on SnZGO and f) O_2_ molecule on SnO_2_, with the inset patterns showing the ELF simulation. ESR spectra of g) DMPO – ·OH; h) DMPO – ·O_2_^−,^ and i) TEMP – ^1^O_2_.

Steady‐state photoluminescence (PL) and time‐resolved PL (TRPL) measurements were conducted to confirm suppressed recombination in SZGO. As shown in **Figure** **6**a,b and Table S5 (Supporting Information), SZGO exhibits lower PL intensity and a shorter carrier lifetime (2.11 ns) than ZGO (2.85 ns), indicating reduced recombination and improved charge mobility.^[^ ^55^ ^]^ Electrochemical impedance spectroscopy (EIS) and transient photocurrent response analyses (Figure 6c,d) further corroborate these results.^[^ ^56^, ^57^ ^]^ SZGO demonstrates higher photocurrent density and a smaller Nyquist arc radius, reflecting improved carrier separation and lower charge‐transfer resistance. Compared to ZGO, SnZGO exhibits improved photoelectric properties due to electron localization, while SnO_2_ loading, forming a heterojunction (SZGO), further enhances carrier separation through directional electron transfer. Thus, that can be expected to enhance the H_2_O and O_2_ molecules adsorption and activation capabilities.

Selective adsorption of H_2_O and O_2_ molecules on SZGO surfaces was investigated using DFT calculations. As shown in Figure 6e and Figure S13a (Supporting Information), H_2_O molecules on SnZGO preferentially interact with Zn sites, while O_2_ molecules bind to doped Sn atoms, slightly elongating their respective bond lengths. Adsorption energies indicate stronger H_2_O binding (E_ads_ = ‐0.58 eV) than O_2_ (E_ads_ = ‐0.22 eV) on SnZGO. In contrast, on SnO_2_, O_2_ molecules form stable interactions with two adjacent Sn atoms (Figure 6f), with a higher adsorption energy (E_ads_ = ‐0.84 eV), while H_2_O exhibits weak physisorption with minimal structural distortion (Figure S13b, Supporting Information). Further simulations show that, in the SZGO composite, H atoms of H_2_O molecules preferentially bond with ZnGa_2_O_4_ domains, whereas O atoms of O_2_ molecules strongly interact with SnO_2_ regions (insets of Figure 6e,f; Figure S13c,d, Supporting Information). These findings confirm spatially selective adsorption of H_2_O and O_2_ on distinct active sites in SZGO, enhancing radical generation by reducing competitive adsorption. In situ spectroscopic analyses, detailed in Section 2.4, further elucidate the spatial adsorption and activation of O_2_ and H_2_O molecules.

Based on the selective adsorption behavior and the regional enrichment of electrons and holes enabled by Sn–O channels construction, the molecular activation mechanism can be summarized as follows: SnO_2_, enriched with photogenerated electrons, demonstrates strong O_2_ adsorption capacity, thereby enhancing superoxide radical formation (O_2_ + e^−^ → ·O_2_^−^).^[^ ^58^ ^]^ Moreover, the ·O_2_^−^ species can further react with holes (h^+^) to generate singlet oxygen (^1^O_2_) via (·O_2_^−^ + h^+^ → ^1^O_2_).^[^ ^59^ ^]^ In contrast, ZnGa_2_O_4_, with its superior H_2_O adsorption capacity, facilitates the activation of H_2_O molecules by photogenerated holes (H_2_O + h^+^ → ·OH).^[^ ^60^ ^]^ This spatial separation of H_2_O and O_2_ on SZGO's ZnGa_2_O_4_ and SnO_2_ domains, driven by surface polarity gradients, minimizes competitive adsorption, enhancing multiple ROS generation. This mechanism maximizes photocatalytic efficiency, as validated by electron spin resonance (ESR) spectroscopy and scavenger experiments.

Subsequently, ESR was employed to identify the ROS generation. In dark condition, TEMPO signals remain stable for both ZGO and SZGO (Figure S14, Supporting Information), indicating negligible electron generation without light excitation. Under UV illumination, TEMPO signal intensity decreases for both samples, with SZGO exhibiting a more pronounced reduction, reflecting enhanced electron generation due to Sn doping and SnO_2_ loading. Furthermore, SZGO shows significantly stronger quartet signals for DMPO‐·OH (1:2:2:1, Figure 6g) and DMPO‐·O_2_^−^ (1:1:1:1, Figure 6h) compared to ZGO, with intensities increasing by 2.14‐ and 3.07‐fold, respectively. A concurrent enhancement of the TEMP‐^1^O_2_ signal (1:1:1, Figure 6i) is also observed. These findings confirm efficient multi‐ROS generation in SZGO. To further validate the active species involved in toluene mineralization, scavenger experiments were conducted using p‐benzoquinone (PBQ, ·O_2_^−^ scavenger), tert‐butanol (TBA, ·OH scavenger), KMnO_4_ (e^−^ scavenger), tryptophan (^1^O_2_ scavenger), and KI (h^+^ scavenger) (Figure S15a,b, Supporting Information). Adding electron or hole scavengers nearly halts photocatalytic activity for both ZGO and SZGO, highlighting the critical role of photoinduced charge carriers.^[^ ^61^ ^]^ In contrast, SZGO maintains relatively high degradation efficiency with individual radical scavengers, indicating the generation of a broad spectrum of ROS. Moreover, the degradation curves and corresponding reaction rate constants derived from radical scavenging experiments (Figure S15c, Supporting Information) reveal distinct temporal contributions of individual ROS. ^1^O; is the primary reactive oxygen species responsible for the rapid toluene mineralization by SZGO. This activity arises from SnO_2_ loading, which induces selective molecular adsorption at active sites and facilitates efficient electron transfer, thereby promoting prolific ^1^O_2_; generation. And the multi‐radical synergistic activation mechanism underpins the superior photocatalytic mineralization performance of SZGO.

These results demonstrate that Sn doping creates electron‐trapping centers, forming Sn─O channels with surface‐loaded SnO_2_, which facilitate directional electron transfer from ZnGa_2_O_4_ to SnO_2_. This enhanced electron mobility suppresses photogenerated charge carrier recombination. Concurrently, spatially selective adsorption of H_2_O and O_2_ molecules maximizes the semiconductor's redox potential by reducing competitive adsorption at active sites. Together, improved charge separation and efficient multi‐ROS generation enable SZGO's exceptional toluene mineralization performance. Furthermore, selective molecular adsorption and activation confer robust adaptability across a wide range of humidity conditions.

### 2.4 Insights from In situ Spectroscopy

**Figure 7:**
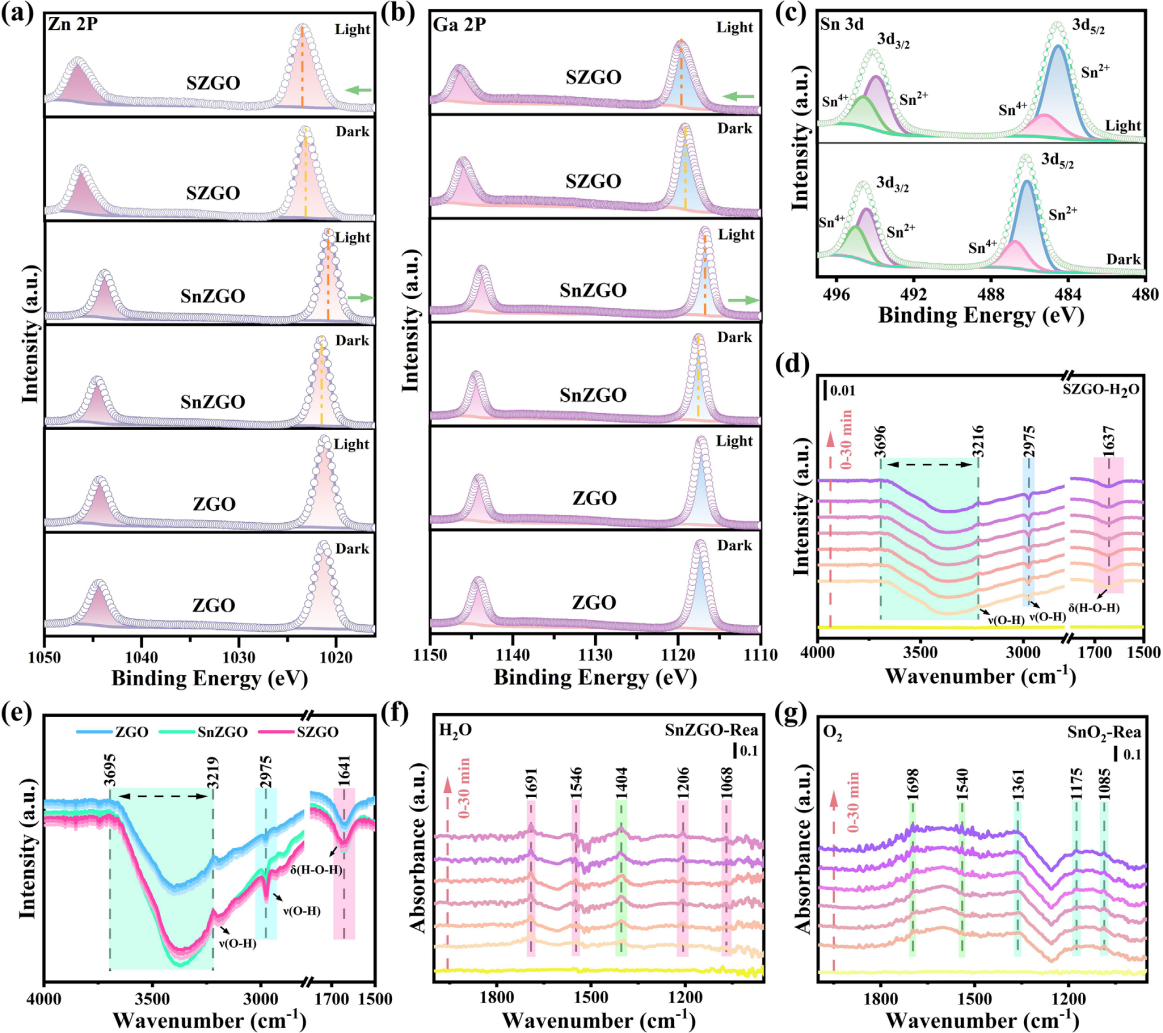
Quasi in situ high‐resolution XPS spectra of a) Zn 2p, b) Ga 2p, and c) Sn 3d under dark and illumination conditions. In situ ATR‐FTIR spectra of H_2_O adsorption on d) SZGO and the corresponding comparison spectra e) on ZGO, SnZGO, and SZGO. In situ DRIFTS spectra of toluene photo‐oxidation on the f) SnZGO under H_2_O‐rich conditions and g) SnO_2_ under O_2_‐rich conditions.

To directly probe charge redistribution and molecular activation during the photocatalytic process, quasi in situ XPS, in situ ATR‐FTIR, and DRIFTS were employed. As shown in **Figure** **7**a,b, ZGO exhibits negligible changes in XPS peak positions before and after illumination, reflecting its limited photoexcited charge redistribution. In contrast, SnZGO displays slight shifts of Zn 2p and Ga 2p peaks toward higher binding energies in dark condition compared to ZGO, while Sn 3d peaks shift toward lower binding energies relative to pure SnO_2_ (Figure 7a,b; Figure S16, Supporting Information). These observations suggest that Sn doping alters the electronic distribution and acts as an electron trapping center within the ZnGa_2_O_4_ lattice. Upon illumination, the Zn 2p and Ga 2p peaks in SnZGO shift back toward lower binding energies, and the Sn 3d peaks shift further downward (Figure 7a,b; Figure S17, Supporting Information), reflecting increased electron density and light‐induced electron accumulation at Sn sites. Peak‐splitting analysis of Sn 3d spectra reveals an increased Sn^2+^ proportion under illumination, confirming the partial reduction of Sn^4+^ by photogenerated electrons (Figure S17, Supporting Information).^[^ ^62^ ^]^ For SZGO, both in dark and illuminated conditions, Zn 2p and Ga 2p peaks consistently shift toward higher binding energies, while Sn 3d peaks shift toward lower binding energies (Figure 7a–c). These trends indicate electron loss from ZnGa_2_O_4_ and electron accumulation on the surface‐loaded SnO_2_, facilitated by Sn–O channels. Notably, a higher proportion of Sn^2+^ is detected in SZGO under illumination (Figure 7c), corroborating efficient electron migration and storage on SnO_2_. Although the O 1s binding energies show minimal shifts (Figure S18, Supporting Information), SZGO exhibits a pronounced decrease in lattice oxygen content and an increase in surface hydroxyl groups after illumination, supporting its superior ability for small‐molecule activation. Overall, these results demonstrate that Sn doping modulates the internal electron distribution of ZnGa_2_O_4_, introduces efficient electron trapping centers, and promotes directional electron transport toward SnO_2_.

In situ ATR‐FTIR, complementing DFT insights in Figure 6e,f, probes H_2_O and O_2_ adsorption behavior on ZGO, SnZGO, and SZGO. As shown in Figure 7d,e and Figure S19a–c (Supporting Information), both SnZGO and SZGO exhibit stronger ν(O─H) stretching (3696–3216 cm^−1^) and δ(H─O─H) bending (1637 cm^−1^) vibrations compared to ZGO, indicating enhanced H_2_O adsorption capacity.^[^ ^63^ ^]^ The progressive intensification of these O─H bands over time further confirms the superior water affinity of SnZGO and SZGO. Notably, the similarity between SnZGO and SZGO in water‐related signals suggests that SnO_2_ loading primarily enhances O_2_ rather than H_2_O adsorption. This is supported by complementary O_2_ adsorption experiments (Figure S20a–d, Supporting Information), where SZGO exhibits more intense vibrational signals than both ZGO and SnZGO, confirming its improved O_2_ uptake. These in situ findings corroborate the DFT‐predicted site‐specific adsorption behavior, wherein H_2_O and O_2_ are preferentially adsorbed on distinct domains—ZnGa_2_O_4_ and SnO_2_, respectively. This spatial separation minimizes competitive adsorption and enables their simultaneous activation. Such spatially resolved molecular interactions significantly facilitate the generation of ROS even at 100% RH, thereby contributing to the superior photocatalytic activity and humidity adaptation of SZGO. More importantly, toluene, as a non‐polar aromatic substance, has been confirmed as mentioned above to be adsorbed in the SnO_2_‐ZnGa_2_O_4_ interface region through π – π interaction, rather than directly competing with H_2_O or O_2_ for the same surface domain. This domain‐specific adsorption minimizes competitive interference and allows simultaneous activation of all three molecules in parallel, enhancing overall photocatalytic efficiency.

To further elucidate the selective adsorption and activation mechanisms, in situ DRIFTS was conducted to monitor the dynamic evolution of toluene species on SnZGO and SnO_2_ surfaces under varying atmospheric conditions. After pretreatment, toluene combined with either H_2_O or O_2_ was introduced into the reaction chamber under 365 nm UV irradiation. Under conditions containing either only H_2_O or only O_2_, SnZGO demonstrates photocatalytic activity toward toluene mineralization (Figure 7f; Figure S21a, Supporting Information). Specifically, in the presence of H_2_O, prominent absorption bands corresponding to H–O–H bending (1640 cm^−1^), C─O stretching vibrations of benzyl alcohol (1206 and 1068 cm^−1^), and C═O stretching vibrations of benzoic acid (1691 and 1546 cm^−1^) progressively intensify over time. Notably, the strong emergence of a formate species (1404 cm^−1^) signal clearly indicates efficient aromatic ring activation and cleavage, highlighting the pivotal role of adsorbed H_2_O in facilitating deep toluene mineralization.^[^ ^64^, ^65^ ^]^ Although similar intermediate species are also detected under O_2_‐only conditions, their vibrational intensities are significantly weaker, confirming that H_2_O adsorption substantially enhances the aromatic ring opening of SnZGO. In comparison, SnO_2_ initially exhibits signals corresponding to benzyl alcohol and benzoic acid in the presence of H_2_O (Figure S21b, Supporting Information). However, these signals diminish progressively over the irradiation period, likely due to accumulation of carbonaceous intermediates that passivate surface active sites, resulting in catalyst deactivation. Conversely, under O_2_‐rich conditions, SnO_2_ shows gradually enhanced intermediate signals (Figure 7g), indicating that O_2_ adsorption effectively maintains active site availability and sustains toluene degradation. Therefore, the above results clearly indicate that ·OH, generated via H_2_O activation, primarily initiate toluene oxidation by attacking the methyl group and promoting ring‐opening, while ·O_2_^−^ and ^1^O_2_, generated via O_2_ activation, synergistically drive the deep oxidation and complete mineralization of the resulting ring‐opened long‐chain intermediates.

**Figure 8:**
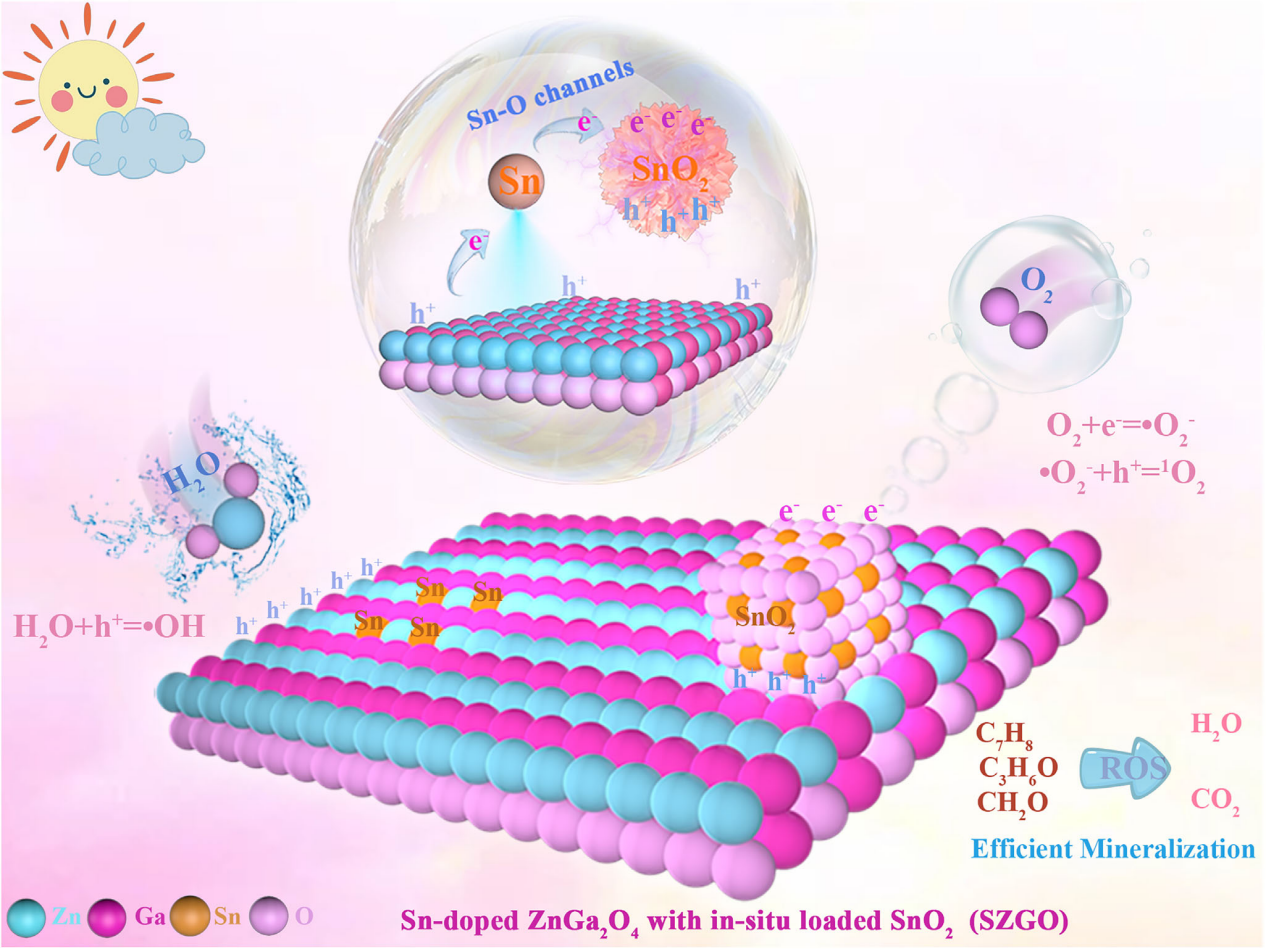
Proposed mechanism for SZGO's efficient photocatalytic VOC degradation.

Collectively, these in situ results confirm that SZGO achieves synergistic photocatalytic performance through spatially selective adsorption and complementary molecular activation. SnZGO domains preferentially adsorb and activate H_2_O molecules, driving aromatic ring opening and deep oxidation, whereas SnO_2_ domains exhibit strong O_2_ affinity, thereby facilitating sustained intermediate oxidation and minimizing carbonaceous residue accumulation. The synergistic integration of polarity‐separated active domains, directional Sn–O electron‐transfer channels, and interfacial substrate pre‐activation allows for efficient spatial coordination of charge carriers, reactant molecules, and ROS. This architecture ensures synchronized generation, activation, and utilization of ROS, ultimately enabling deep mineralization of toluene under ambient conditions (**Figure** **8**).

## 3 Conclusion

In conclusion, we propose an in situ construction strategy to synthesize Sn‐doped ZnGa_2_O_4_ integrated with surface‐loaded SnO_2_ nanoparticles (SZGO). The doped Sn atoms act as electron trapping centers, effectively suppressing the recombination of photogenerated electrons and holes in ZnGa_2_O_4_. Moreover, the formation of unique Sn–O channels between the doped Sn atoms and the oxygen atoms of SnO_2_ facilitates directional electron transfer, significantly enhancing electron migration efficiency and minimizing electron loss. Importantly, spatially selective molecular adsorption is achieved: the Sn‐doped ZnGa_2_O_4_ domains preferentially adsorb H_2_O molecules, promoting the generation of ·OH radicals that drive aromatic ring‐opening, while the SnO_2_ domains, enriched in electrons, exhibit stronger O_2_ adsorption, favoring the formation of ·O_2_^−^ and ^1^O_2_ species for the degradation of carbonaceous intermediates. This site‐specific adsorption enables efficient and simultaneous activation of multiple ROS, ultimately leading to nearly 100% toluene degradation and 99.2% mineralization efficiency. Moreover, the separated adsorption strategy imparts SZGO with exceptional adaptability under a wide range of humidity conditions. By addressing charge recombination and competitive adsorption, this study offers a robust framework for gas‐phase pollutant photocatalysis, paving the way for advanced catalyst design.

## Conflict of Interest

The authors declare no conflict of interest.

## Supporting information



Supporting Information

## Data Availability

The data that support the findings of this study are available in the supplementary material of this article.

## References

[1] Q. Shen , J. Chen , X. Jing , C. Duan , Angew. Chem., Int. Ed. 2025, 10.1002/ange.202506277. 40372156

[2] X. Dong , W. Cui , H. Wang , J. Li , Y. Sun , H. Wang , Y. Zhang , H. Huang , F. Dong , Sci. Bull 2019, 64, 669. 10.1016/j.scib.2019.04.020 36659649

[3] J. Yuan , G. Li , X. Liu , Y. Yang , F. Yu , J. Cao , Z. Fei , J. Ma , M. Nazeeruddin , P. Dyson , Adv. Funct. Mater. 2024, 34, 2401281.

[4] X. Qiu , Y. Sang , H. Wu , X. Xue , Z. Yan , Y. Wang , Z. Cheng , X. Wang , H. Tan , S. Song , G. Zhang , X. Zhang , N. Houk , N. Jiao , Nature 2021, 597, 64. 34280952 10.1038/s41586-021-03801-y

[5] H. Ma , X. Hu , X. Wang , W. Xu , Y. Shen , R. Fang , Y. Li , Y. Liu , F. Dong , Appl. Catal. B‐Enviro. 2025, 361, 124638.

[6] H. Jin , P. Zhao , Y. Qian , J. Xiao , Z. Chao , H. Jiang , Chem. Soc. Rev. 2024, 53, 9378. 39163028 10.1039/d4cs00095a

[7] Y. Guo , M. Wen , S. Song , Q. Liu , G. Li , T. An , Appl. Catal. B‐Enviro. 2022, 308, 121212.

[8] Y. Zhang , Y. Wang , R. Xie , B. Huang , M. Leung , J. Li , D. Leung , Environ. Sci. Technol. 2022, 56, 16582. 36367480 10.1021/acs.est.2c05444

[9] Z. Chen , Y. Peng , J. Chen , C. Wang , H. Yin , H. Wang , C. You , J. Li , Environ. Sci. Technol. 2020, 54, 14465. 33119280 10.1021/acs.est.0c06048

[10] X. Cao , Z. Chen , R. Lin , W. Cheong , S. Liu , J. Zhang , Q. Peng , C. Chen , T. Han , X. Tong , Y. Wang , R. Shen , W. Zhu , D. Wang , Y. Li , Nat. Catal. 2018, 1, 704.

[11] Y. Luo , X. Wang , F. Gao , L. Jiang , D. Wang , H. Pan , Adv. Funct. Mater. 2024, 33, 2418427.

[12] Q. Liu , D. Wu , Y. Zhou , H. Su , R. Wang , C. Zhang , S. Yan , M. Xiao , Z. Zou , ACS Appl. Mater. Interfaces 2014, 6, 2356. 24475972 10.1021/am404572g

[13] Y. Chai , S. Tang , Q. Wang , Q. Wu , J. Liang , L. Li , Appl. Catal. B‐Enviro. 2023, 338, 123012.

[14] J. Liang , Y. Chai , L. Li , D. Li , J. Shen , Y. Zhang , X. Wang , Appl. Catal. B‐Enviro. 2020, 265, 118551.

[15] M. Xi , X. Yu , X. Su , L. Xiong , X. Ning , P. Gao , Z. Huang , C. Chang , ACS Catal. 2025, 15, 4185.

[16] Z. Yang , Y. Shi , H. Li , C. Mao , X. Wang , X. Liu , X. Liu , L. Zhang , Environ. Sci. Technol. 2022, 56, 3587. 35199995 10.1021/acs.est.1c08532

[17] X. Liu , M. Wang , H. Yin , J. Hu , K. Cheng , J. Kang , Q. Zhang , Y. Wang , ACS Catal. 2020, 10, 8303.

[18] X. Zhang , J. Huang , K. Ding , Y. Hou , X. Wang , X. Fu , Environ. Sci. Technol. 2009, 43, 5947. 19731702 10.1021/es900403a

[19] D. Yang , Y. Guo , Z. Yu , Z. Jiang , W. Xiang , X. Wu , J. Wang , Environ. Sci. Technol. 2025, 59, 7117. 40173186 10.1021/acs.est.4c14436

[20] Y. Li , B. Chen , L. Liu , B. Zhu , D. Zhang , Angew. Chem., Int. Ed. 2024, 63, 202319432. 10.1002/anie.202319432 38233346

[21] W. Jin , C. Yang , R. Pau , Q. Wang , E. Tekelenburg , H. Wu , Z. Wu , S. Jeong , F. Pitzalis , T. Liu , Q. He , Q. Li , J. Huang , R. Kroon , M. Heeney , H. Woo , A. Mura , A. Motta , A. Facchetti , M. Fahlman , M. Loi , S. Fabiano , Nature 2024, 630, 96. 38750361 10.1038/s41586-024-07400-5 PMC11153156

[22] S. He , Y. Chen , J. Fang , Y. Liu , Z. Lin , Chem. Soc. Rev. 2025, 54, 2154. 39838850 10.1039/d4cs00317a

[23] B. Moss , Q. Wang , K. Butler , R. Grau‐Crespo , S. Selim , A. Regoutz , T. Hisatomi , R. Godin , D. Payne , A. Kafizas , K. Domen , L. Steier , J. Durrant , Nat. Mater. 2021, 20, 511. 33432143 10.1038/s41563-020-00868-2

[24] X. Zhang , F. Bi , Z. Zhu , Y. Yang , S. Zhao , J. Chen , X. Lv , Y. Wang , J. Xu , N. Liu , Appl. Catal. B‐Enviro. 2021, 297, 120393.

[25] W. Wang , W. Zhang , C. Deng , H. Sheng , J. Zhao , Angew. Chem., Int. Ed. 2024, 136, 202317969. 10.1002/anie.202317969 38155103

[26] G. Ren , M. Zhou , P. Hu , J. Chen , H. Wang , Nat. Commun. 2024, 15, 2346. 38490989 10.1038/s41467-024-46749-z PMC10943107

[27] E. Bouteh , M. Bentel , E. Cates , J. Hazard. Mater. 2023, 453, 131437. 37086670 10.1016/j.jhazmat.2023.131437

[28] B. Zhang , Y. Shen , B. Liu , J. Ji , W. Dai , P. Huang , D. Zhang , G. Li , R. Xie , H. Huang , Environ. Sci. Technol. 2023, 57, 7041. 37078822 10.1021/acs.est.2c08867

[29] J. Zhang , G. Chen , Q. Liu , C. Fan , D. Sun , Y. Tang , H. Sun , X. Feng , Angew. Chem., Int. Ed. 2022, 61, 202209486. 10.1002/anie.202209486 PMC9804859 35862112

[30] Z. Lin , J. Liu , S. Li , J. Liang , X. Liu , L. Xie , G. Lu , J. Han , Y. Huang , Q. Li , Adv. Funct. Mater. 2023, 33, 2211638.

[31] B. Chen , Y. Duan , S. Chen , Y. Li , Y. Zhu , ACS Catal. 2024, 14, 3966.

[32] W. Dai , S. Zhang , H. Shang , S. Xiao , Z. Tian , W. Fan , X. Chen , S. Wang , W. Chen , D. Zhang , Adv. Funct. Mater. 2024, 34, 2309426

[33] J. Kong , Y. Sun , X. Ge , M. Mao , H. Yu , Y. Wang , Adv. Funct. Mater. 2023, 33, 2209579.

[34] Y. Yang , J. Zhou , L. Zhou , H. Li , R. Xie , X. Zeng , Y. Liu , Y. Zhi , S. Shan , K. Yao , Adv. Funct. Mater. 2025, 10.1002/adfm.202425346.

[35] Y. Liu , X. Chen , M. Kamali , B. Rossi , L. Appels , R. Dewil , Adv. Funct. Mater. 2024, 34, 2405741.

[36] J. He , S. Yang , X. Xiao , D. Fang , R. Miao , C. Wang , L. Chen , N. Li , J. Li , Y. Su , H. Jin , Energy Storage Mater. 2025, 75, 104037.

[37] A. Treglia , A. Olivati , V. Romano , A. Iudica , G. M. Paternò , I. Poli , A. Petrozza , Adv. Energy Mater. 2025, 15, 2404905.

[38] Q. Guan , W. Ran , D. Zhang , W. Li , N. Li , B. Huang , T. Yan , Adv. Sci. 2024, 11, 2401990. 10.1002/advs.202401990 PMC11321682 38868931

[39] B. Chen , Y. Duan , J. Tan , Y. Li , S. Li , D. Zhang , Adv. Funct. Mater. 2025, 35, 2425956.

[40] J. Yu , X. Wang , L. Chen , G. Lu , G. Shi , X. Xie , Y. Wang , J. Sun , Chem. Eng. J. 2022, 435, 135033.

[41] G. Zhou , B. Lei , F. Dong , ACS Catal. 2024, 14, 4791.

[42] H. Zhang , S. Liu , A. Zheng , P. Wang , Z. Zheng , Z. Wang , H. Cheng , Y. Dai , B. Huang , Y. Liu , Angew. Chem., Int. Ed. 2024, 63, 202400965. 10.1002/anie.202400965 38363034

[43] Y. Chen , S. Ke , X. Yang , L. Shen , M. Yang , Adv. Funct. Mater. 2025, 35, 2419519.

[44] Y. Zhang , H. Zhao , S. Jiang , Y. Zhang , Y. Chen , J. Gong , Adv. Sci. 2025, 12, 2413379. 10.1002/advs.202413379 PMC12079335 40091518

[45] S. Du , S. Lin , K. Ren , C. Li , F. Zhang , Appl. Catal. B‐Enviro. 2023, 328, 122503.

[46] X. Song , H. Ma , Y. Li , Y. Duan , W. Wang , F. Dong , Appl. Catal. B‐Enviro. 2025, 365, 124955.

[47] Y. Huang , X. Zhang , L. Li , M. Humayun , H. Zhang , X. Xu , S. Anthony , Z. Chen , J. Zeng , D. Shtansky , K. Huo , H. Song , C. Wang , W. Zhang , Adv. Funct. Mater. 2025, 35, 2401011.

[48] L. Wang , S. Liu , Z. Liu , M. Han , J. Tian , Y. Xiao , Q. Chen , D. Hu , L. Zhang , L. Kang , Q. Dai , Adv. Mater. 2025, 37, 2418230. 10.1002/adma.202418230 40259561

[49] L. Liccardo , M. Bordin , P. Sheverdyaeva , M. Belli , P. Moras , A. Vomiero , E. Moretti , Adv. Funct. Mater. 2023, 33, 2370138.

[50] G. Ou , Y. Xu , B. Wen , R. Lin , B. Ge , Y. Tang , Y. Liang , C. Yang , K. Huang , D. Zu , R. Yu , W. Chen , J. Li , H. Wu , L. Liu , Y. Li , Nat. Commun. 2018, 9, 1302. 29615620 10.1038/s41467-018-03765-0 PMC5882908

[51] N. Xiao , S. Li , X. Li , L. Ge , Y. Gao , N. Li , Chin. J. Catal. 2020, 41, 642.

[52] H. Yu , J. Huang , L. Jiang , L. Leng , K. Yi , W. Zhang , C. Zhang , X. Yuan , Appl. Catal. B‐Enviro. 2021, 298, 120618.

[53] A. Meng , L. Zhang , B. Cheng , J. Yu , Adv. Mater. 2019, 31, 1807660. 10.1002/adma.201807660 31148244

[54] S. Wu , P. Schmuki , Adv. Mater. 2025, 37, 2414889. 39969405 10.1002/adma.202414889 PMC11837903

[55] Z. Shen , Z. Luo , J. Chen , Y. Li , Adv. Funct. Mater. 2023, 33, 2213935.

[56] T. Xue , L. Chen , K. Li , B. Lei , H. Wang , F. Dong , Y. Yang , Environ. Sci. Technol. 2023, 57, 8174. 37199463 10.1021/acs.est.3c00103

[57] L. Yang , Z. Chen , Q. Cao , H. Liao , J. Gao , L. Zhang , W. Wei , H. Li , J. Lu , Adv. Mater. 2024, 36, 2306758. 10.1002/adma.202306758 37865887

[58] J. Li , J. Wang , S. Shen , R. Chen , M. Liu , F. Dong , Environ. Sci. Technol. 2023, 57, 5445. 36942694 10.1021/acs.est.2c09669

[59] S. Wang , W. Cui , B. Lei , X. Dong , Y. Tang , F. Dong , Environ. Sci. Technol. 2023, 57, 12890. 37590166 10.1021/acs.est.3c03396

[60] X. Xia , J. Feng , Z. Zhong , X. Yang , N. Li , D. Chen , Y. Li , Q. Xu , J. Lu , Adv. Funct. Mater. 2024, 34, 2311987.

[61] J. Liang , H. Zhang , Q. Song , Z. Liu , J. Xia , B. Yan , X. Meng , Z. Jiang , X. Wen , D. Lou , C. Lee , Adv. Mater. 2024, 36, 2303287. 10.1002/adma.202303287 37973198

[62] B. Li , J. Chen , L. Wang , D. Xia , S. Mao , L. Xi , S. Ying , H. Zhang , Y. Wang , Appl. Catal. B‐Enviro. 2025, 363, 124784.

[63] X. Dong , Z. Cui , Y. Sun , F. Dong , ACS Catal. 2021, 11, 8132.

[64] M. Volokh , M. Shalom , Nat. Catal. 2021, 4, 350.

[65] J. Zhang , B. Shen , Z. Hu , M. Zhen , S. Guo , F. Dong , Appl. Catal. B‐Enviro. 2021, 296, 120376.

