# Supplementary material for: Spatially Separated Molecular Activation Over Dual Sites in Sn‐Doped ZnGa2O4/SnO2 Photocatalysts Toward Complete VOCs Mineralization
Source: Adv Sci (Weinh). 2025 Aug 22;12(40):e09891. doi: 10.1002/advs.202509891 (PMC12561370; doi:10.1002/advs.202509891)
Supplement: Supplementary file 1 — Supporting Information [file ADVS-12-e09891-s001.docx]

**Supporting Information**

**Spatially Separated Molecular Activation over Dual Sites in Sn-Doped ZnGa_2_O_4_/SnO_2_ Photocatalysts toward Complete VOCs Mineralization**

Xianli Hu,^a^ Bin Liu,^a^ Hao Ma,^a*^ Ruimei Fang,^a^ Ting Zhang,^a^ Yuhan Li,^a^ Behzad Rezaei,^c^ Fan Dong ^b*^

^a^ National Research Base of Intelligent Manufacturing Service, College of Environment and Resources, Chongqing Technology and Business University, Chongqing 400067, China.

^b^ Research Center for Carbon-Neutral Environmental & Energy Technology, Institute of Fundamental and Frontier Sciences, University of Electronic Science and Technology of China, Chengdu 611731, China.

^c^ Department of Chemistry, Isfahan University of Technology, Isfahan, 84156–83111, Iran.

E-mail: mahaoke@126.com (Hao Ma); dfctbu@126.com (Fan Dong)

# S1. Preparation of Catalysts

All chemical reagents used in this study were of analytical grade and employed without further purification. The catalysts were synthesized via a one-step hydrothermal method. Specifically, Ga(NO_3_)_3_·9H_2_O (5 mmol), SnCl_4_·5H_2_O (3.75 mmol), and Zn(NO_3_)_2_·5H_2_O (2.5 mmol) were dissolved in 50 mL of deionized water under continuous stirring for 30 minutes. Subsequently, a mixed solution of deionized water and concentrated ammonia (H_2_O: NH_3_ = 3:1, v/v) was added dropwise to adjust the pH to 8. After an additional 30 minutes of stirring, the resulting suspension was transferred into a 200 mL Teflon-lined stainlesssteel autoclave and maintained at 160 °C for 24 h.

Upon completion of the hydrothermal reaction, the autoclave was cooled to room temperature naturally. The resulting precipitate was collected by centrifugation and washed alternately with deionized water and ethanol six times to remove residual ions. The purified product was then dried at 60 °C in an oven and ground into a fine powder to obtain Sn-doped ZnGa_2_O_4_ with in-situ formed SnO_2_ nanoparticles. These samples are denoted as SZGO-x, where x corresponds to the relative molar ratio of SnCl_4_·5H_2_O used in the precursor solution. Among them, the sample with the optimized performance, SZGO-3, exhibited the highest oxidative capacity and was selected as the representative photocatalyst for further investigation.

For comparison, a pristine ZnGa_2_O_4_ sample (ZGO) was synthesized using the same procedure but without the addition of SnCl_4_·5H_2_O. A pure SnO_2_ sample was also obtained by adding only SnCl_4_·5H_2_O to the precursor solution under identical conditions. To isolate the role of Sn doping, a Sn-doped ZnGa_2_O_4_ sample (SnZGO) was prepared by reducing the dosage of SnCl_4_·5H_2_O to inhibit the formation of SnO_2_ nanoparticles. Additionally, a physical mixture of SnO_2_ and ZnGa_2_O_4_ (SnO_2_/ZGO) was obtained by grinding the individual SnO_2_ and ZGO powders in a molar ratio equivalent to that of SZGO-3 until fully homogenized. The exact reagent dosages for all samples are detailed in Table S1.

# S2. Experimental Procedures

The photocatalytic activity, stability, and selectivity of the prepared catalysts were evaluated using a continuous-flow reactor with dimensions of 200 mm × 100 mm × 17 mm, as illustrated in Scheme S1. For each test, 0.05 g of the catalyst powder was uniformly dispersed on a glass disk pre-moistened with deionized water. A total of four such disks were prepared, dried at 60 °C, and subsequently placed into the reaction chamber. The target gas, consisting of toluene mixed with both dry and humidified air (relative humidity of 50%), was introduced into the reactor at a total flow rate of 1 L·min^-1^. After establishing adsorption–desorption equilibrium (toluene concentration stabilized at ~60 ppm), photocatalytic degradation experiments were initiated under ultraviolet (UV) light irradiation using a 150 W high-pressure mercury lamp (λ < 365 nm) as the light source. The concentrations of toluene (C_7_H_8_), H_2_O, and CO_2_ in the outlet gas were continuously monitored using an online photoacoustic multi-gas analyzer (GASERA ONE, Finland). Based on the measured concentrations, the contaminant removal efficiency and mineralization efficiency were calculated using the following equations:

$\eta\left( \% \right)=\frac{C_{0}-C}{C_{0}}\times100\%$ (1)

$M\left( \% \right)=\frac{C_{out}}{C_{0}\times n}\times100\%$ (2)

Where C_0_ is the initial concentration of the target contaminant (toluene) at adsorption equilibrium before irradiation; C is the real-time concentration of the contaminant after light irradiation; C_out_ is the real-time concentration of CO₂ in the outlet gas; n is the number of carbon atoms in a toluene molecule (n = 7).

The pseudo-first-order rate constant k for the photocatalytic reaction was determined using the following equation:

$k=\frac{1}{t}\times ln(\frac{c_{0}}{c})$ (3)

Where C_0_ and C are as defined above; t is the reaction time (min).

# S3. Sample Characterizations

The crystalline phase compositions of the photocatalysts were characterized by X-ray diffraction (XRD) using a Bruker D8 diffractometer. The surface morphology and microstructure were examined by scanning electron microscopy (SEM, JEOL JSM-6490, Japan) and transmission electron microscopy (TEM, JEM-2010, Japan), respectively. X-ray photoelectron spectroscopy (XPS) analysis was conducted using a Thermo Fisher Scientific ESCALAB 250Xi spectrometer, with all binding energies calibrated to the C 1s peak at 284.8 eV. Steady-state photoluminescence (PL) spectra were recorded on a Hitachi F-4600 fluorescence spectrophotometer, while time-resolved photoluminescence (TRPL) spectra were measured using an FLS1000 fluorescence spectrometer (Edinburgh Instruments, UK) with an excitation wavelength of 450 nm. Electron paramagnetic resonance (EPR) spectroscopy was employed to detect oxygen vacancies and reactive oxygen species (ROSs), using a continuous-wave X-band spectrometer (EPR200-Plus, China Instruments & QuantumTech, Hefei, China). Photoelectrochemical properties of the samples were evaluated on a CHI 760E electrochemical workstation (Chenhua, Shanghai) in a standard three-electrode configuration, consisting of the catalyst-coated working electrode, a platinum wire counter electrode, and an Ag/AgCl reference electrode. Photocurrent measurements were carried out under UV irradiation using a 3 W LED light source (λ = 365 ± 10 nm, Shenzhen LAMPLIC), with 0.4 M Na_2_SO_4_ aqueous solution as the electrolyte. Electrochemical impedance spectroscopy (EIS) and Mott–Schottky analyses were also performed using the same workstation to assess the charge-transfer resistance and semiconductor properties of the photocatalysts. The optical absorption properties were investigated by UV–vis diffuse reflectance spectroscopy (DRS) using a Shimadzu UV-2550 spectrophotometer. The CB and VB values derived from UV–vis DRS and Mott–Schottky analysis are referenced against the reversible hydrogen electrode (RHE) (ECB vs. SHE = – (EM–SCE + 0.24 V) at 25 °C), which closely approximates the standard hydrogen electrode (SHE/NHE) at pH = 0.

# S4. In-Situ Characterizations

The synthesized catalysts were further investigated using quasi in-situ XPS equipped with a 150 W mercury lamp to monitor dynamic changes in surface chemical states during photocatalytic reactions. XPS measurements were performed before and after UV irradiation, allowing real-time observation of element-specific valence state transitions and surface redox behavior. Upon light exposure, noticeable shifts in the binding energies of core-level peaks were observed, indicating photoinduced charge separation and modulation of surface active sites. In particular, the Sn 3d spectra exhibited distinct changes in the valence states of Sn species under illumination, providing strong evidence for the formation of electron-trapping centers and the establishment of efficient electron transport pathways. These observations underscore the crucial role of Sn-related sites in mediating interfacial charge dynamics and enhancing photocatalytic activity.

To investigate the adsorption configuration and active sites of H_2_O and O_2_ molecules on the photocatalyst surface, in-situ attenuated total reflection Fourier-transform infrared (ATR-FTIR) spectroscopy was carried out using a ConcentratIR™ 2 (Harrick) accessory coupled with a Bruker Vertex 70V spectrometer equipped with a mercury cadmium telluride (MCT) detector (Scheme S2). The fresh catalyst powder was first dispersed in deionized water, and a drop of the resulting suspension was deposited onto the diamond ATR crystal. The sample was then dried in vacuum to remove excess moisture and ensure uniform adherence to the crystal surface. Initially, the sample was subjected to ultra-high vacuum conditions (~2 × 10^-5^ Pa) to eliminate residual impurities and pre-adsorbed H_2_O molecules. Subsequently, water vapor and argon were introduced into the chamber until atmospheric pressure was reached, enabling controlled H_2_O adsorption. For O_2_ adsorption experiments, pure oxygen was introduced into the reactor instead of water vapor.

In-situ diffuse reflectance infrared Fourier transform spectroscopy (DRIFTS) was employed to monitor the evolution of surface-adsorbed intermediate species during the adsorption and photocatalytic reaction processes, as illustrated in Scheme S3. The measurements were conducted using a Bruker Tensor II FTIR spectrometer equipped with a specialized DRIFTS reaction chamber featuring three optical ports. Infrared radiation passed through two ZnSe windows, while a quartz window enabled the transmission of UV light to initiate the photocatalytic reaction. The reaction cell was equipped with both gas inlet and outlet ports, and its temperature was precisely controlled via a circulating cooling water system and thermocouple feedback. Prior to measurements, the catalyst samples were pretreated by heating at 110 °C under flowing helium for 30 minutes to remove adsorbed impurities and moisture. The background IR spectrum of the sample was then recorded under helium as a reference. Subsequently, a mixed gas stream containing O_2_, H_2_O vapor, and toluene was introduced into the chamber, and IR spectra were collected at 2-minute intervals to capture the adsorption dynamics. After establishing adsorption equilibrium, the catalyst was exposed to UV light irradiation for 30 minutes, during which IR spectra were continuously recorded every 2 minutes. The scanning range of 4000–600 cm^-1^ was used to identify vibrational signals associated with adsorbed species and intermediates. As detailed in Section 2.4, in-situ DRIFTS provided real-time insights into the dynamic surface evolution of toluene on SnZGO and SnO_2_ under different conditions. By selectively introducing either O_2_ or H_2_O, key reaction intermediates were successfully identified under photocatalytic conditions, enabling mechanistic interpretation of the oxidation pathway.

# S5. Density Functional Theory (DFT) calculations

To gain mechanistic insights into the influence of Sn doping and SnO_2_ loading on the electronic structure and photocatalytic behavior of ZnGa_2_O_4_, density functional theory (DFT) calculations were performed to investigate the energy band structure, electron localization function (ELF), molecular activation of H_2_O and O_2_, and interfacial charge transfer processes. All spin-polarized DFT calculations were carried out using the Vienna Ab initio Simulation Package (VASP) with the Perdew–Burke–Ernzerhof (PBE) exchange-correlation functional under the generalized gradient approximation (GGA) and the projector augmented wave (PAW) method. A plane-wave cutoff energy of 400 eV was applied in all calculations. The Brillouin zone was sampled using a Monkhorst–Pack k-point mesh of 3 × 3 × 1. Geometry optimizations were performed until the forces on each atom were below 0.03 eV·Å^-1^ and the total energy convergence threshold was 10^-6^ eV.

In this study, a ZnGa_2_O_4_ (311) slab model was constructed to simulate the catalyst surface. During structure optimization, the bottom layers of each slab were fixed to their bulk positions, while the upper layers were fully relaxed. A vacuum layer of 15 Å was introduced along the surface normal direction to eliminate spurious interactions between periodic images.

1)Model construction rationale

For Sn-doped ZnGa_2_O_4_ (SnZGO), we constructed a 2 × 1 × 1 ZnGa_2_O_4_ supercell, in which one Zn^2+^ or Ga^3+^ was replaced with a Sn^4+^ atom.

Substitution at the Zn site was chosen as the primary model because formation energy calculations showed this pathway is thermodynamically more favorable, consistent with experimental evidence from XPS and literature reports.

2) Heterostructure simulation

The SZGO interface model was generated by stacking the (311) surface of ZnGa_2_O_4_ with the (110) surface of SnO_2_, aligned based on minimal lattice mismatch. Interfacial Sn–O bonding was introduced to reflect the experimental in-situ growth of SnO_2_ on SnZGO.

3) Assumptions and limitations

All simulations were conducted under vacuum conditions, without explicitly modeling temperature or solvent (e.g., water vapor) effects. While oxygen vacancies were detected via ESR, they were not included in the slab models due to computational cost and the focus on intrinsic adsorption behavior. Charge distribution analysis (charge density difference) was used to approximate electron transfer trends.

The adsorption energy (E_ads_) of H_2_O, O_2_, and toluene molecules on each catalyst surface was calculated using the following equation:

E_ads_ = E_tot_ – E_molecule_ – E_catalyst_ (4)

Where E_tot_ is the total energy of the adsorption system; E_molecule_ is the energy of the isolated gas-phase molecule (H_2_O, O_2_, or toluene); E_catalyst_ is the total energy of the relaxed catalyst model (ZGO, SnZGO, or SZGO).

# Tables

**Table S1 List of parameters for drug dosage.**

| **Sample** | **Zn(NO_3_)_2_·5H_2_O (mmol)** | **Ga(NO_3_)_3_·9H_2_O (mmol)** | **SnCl_4_·5H_2_O (mmol)** |
| --- | --- | --- | --- |
| **ZGO** | 2.5 | 5 mmol | \ |
| **SnO_2_** | \ | \ | 5 |
| **SnZGO** | 2.5 | 5 | 0.16 |
| **SZGO-1** | 2.5 | 5 | 1.25 |
| **SZGO-2** | 2.5 | 5 | 2.5 |
| **SZGO-3 (SZGO)** | 2.5 | 5 | 3.75 |
| **SZGO-4** | 2.5 | 5 | 5 |
| **SZGO-5** | 2.5 | 5 | 6.25 |

**Table S2 List of parameters for BET tests.**

| **Sample** | **BET Surface Area (m^2^/g**) | **Desorption Average Pore Diameter (nm)** |
| --- | --- | --- |
| ZGO | 126.41 | 10.37 |
| SZGO | 156.66 | 3.53 |

**Table S3 Toluene degradation of various photocatalysts.**

| **Photocatalysts** | **Light Source** | **Degradation Efficiency** | **Mineralization** | **Relative**  **Humidity** | **Degradation Efficiency** | **Cycle Tests** | **Years** | **Ref.** |
| --- | --- | --- | --- | --- | --- | --- | --- | --- |
| TiO_2_ | UV/VUV | 50% | 65% | 0-85% | 23-48% (N_2_+O_2_) | 1 h | 2020 | [1] |
| TiO_2_@UiO-66 | UV light | 66.59% | 89% | 0-80% | 66.59% (RH=60%) | 9 h | 2020 | [2] |
| α-Ga_2_O_3_/MgAl-LDH | An UV high-pressure mercury lamp (300 W, λ< 365 nm) | 90.71% | 84.0% | 5-90% | More than 90% | 3 h | 2020 | [3] |
| 2-Zn-SnO_2_ |  | 77.5% | / | 50%, 100% | 77.5% | 4 h | 2021 | [4] |
| Zn-Ti-LDHs | A 300 W mercury lamp | 75.2% | 83% | 10-90% | 55%-71.4% | 3 h | 2022 | [5] |
| SnO_2_/ZnSn(OH)_6_ | A 300 W Hg lamp | 72.1% | 81.1% | 0%, 50% | 68.5%, 70.1% | 7 h | 2023 | [6] |
| LDH/ZSO-75 | An UV high-pressure mercury lamp (300 W, λ< 365 nm) | 89.8% | 83.1% | 60% | 89.8% | 3h | 2023 | [7] |
| ZnSnAl-LDH | An ultraviolet (UV) light (254 nm) | 81% | CO_2_ concentration (117 ppm) | 0-75% | Over 81% | 8 h | 2024 | [8] |
| AZHS-20 | An UV high-pressure mercury lamp (300 W, λ< 365 nm) | 94.25% | 101.8% | 50-75% | 94.25% | 10 h | 2024 | [9] |
| UN-300@TiO_2_ | A 300 W xenon lamp | 86.2% | 90.08% | 0-75% | 86.2-89.86% | 400 min | 2025 | [10] |
| ZBSO | An UV high-pressure mercury lamp (300 W, λ< 365 nm) | 91.1% | 98.8% | 0-75% | 85.5-91.1% | 5 h | 2025 | [11] |
| **SZGO** |  | **nearly 100%** | **99.2%** | **0-100%** | **98.15-100%** | **12 h** | **2025** | **This work** |

**Table S4 The adsorption and photocatalytic oxidation species of toluene on ZGO and SZGO by in-situ DRIFTS.**

|  | **ZGO** | | | **SZGO** | | |
| --- | --- | --- | --- | --- | --- | --- |
|  | **Wavenumber (cm^-1^)** | **Band Attribution** | **Ref.** | **Wavenumber (cm^-1^)** | **Band Attribution** | **Ref.** |
| Adsorption mode | 3670-3000 | ν(O-H) | [12] | 3670-3000 | ν(O-H) | [12] |
|  | 2930 | C-H bond of C_7_H_8_ | [13] | 2935 | C-H bond of C_7_H_8_ | [13] |
|  | 1649 | H_2_O | [14] | 1649 |  | [14] |
|  | 1628 | skeletal C-C bond of C_7_H_8_ | [15] | 1628 | skeletal C-C bond of C_7_H_8_ | [15] |
|  | 1608 | C=C bond of benzene ring | [16, 17] | 1611 | C=C bond of benzene ring | [16, 17] |
|  | 1370 | benzyl alcohol | [18] | 1370 | benzyl alcohol | [11] |
|  | 1025 | benzyl alcohol | [19] | 1256 | benzyl alcohol | [18] |
| Reaction mode | 3670-3000 | ν(O-H) | [12] | 3670-3000 | ν(O-H) | [12] |
|  | 2930 | C-H bond of C_7_H_8_ | [13] | 2935 | C-H bond of C_7_H_8_ | [13] |
|  | 1747 | benzaldehyde | [20, 21] | 1793 | benzaldehyde | [19, 20] |
|  | 1554 | benzyl alcohol | [22] | 1732 | benzaldehyde | [23] |
|  | 1358 | hydrocarbon mixture | [16, 17] | 1701 | benzoic acid | [22] |
|  | 1351 | benzyl alcohol | [15] | 1691 | benzoic acid | [25] |
|  | 1351 | benzyl alcohol | [15] | 1652 | benzyl alcohol | [18] |
|  | 1208 | benzyl alcohol | [26] | 1599 | benzoic acid | [15] |
|  | 1025 | benzyl alcohol | [19] | 1554 | benzoic acid | [23] |
|  |  |  |  | 1494 | benzoic acid | [24] |
|  |  |  |  | 1404 | formic acid | [27,28] |
|  |  |  |  | 1351 | benzyl alcohol | [15] |
|  |  |  |  | 1208 | benzyl alcohol | [25] |
|  |  |  |  | 1181 | benzyl alcohol | [26] |
|  |  |  |  | 1025 | benzyl alcohol | [18] |

**Table S5 List of parameters for time-resolved photoluminescence tests.**

| **Sample** | **A_1_** | **τ_1_ (ns)** | **A_2_** | **τ_2_ (ns)** | **τ_Ave_ (ns)** |
| --- | --- | --- | --- | --- | --- |
| ZGO | 39.96 | 1.54 | 60.04 | 6.56 | 2.85 |
| SZGO | 43.73 | 1.18 | 56.27 | 5.45 | 2.11 |

**Table S6 Adsorbed species of H_2_O identified by in-situ ATR-FTIR.**

| **Wavenumber (cm^-1^)** | **Peak Attribution** | **Ref.** |
| --- | --- | --- |
| 3696 | ν(O-H) | [29] |
| 3664 | ν(O-H) | [29,30] |
| 3216 | ν(O-H) | [30,31] |
| 2920 | ν(O-H) | [30] |
| 1637 | δ(H-O-H) | [29, 12] |

# Schemes


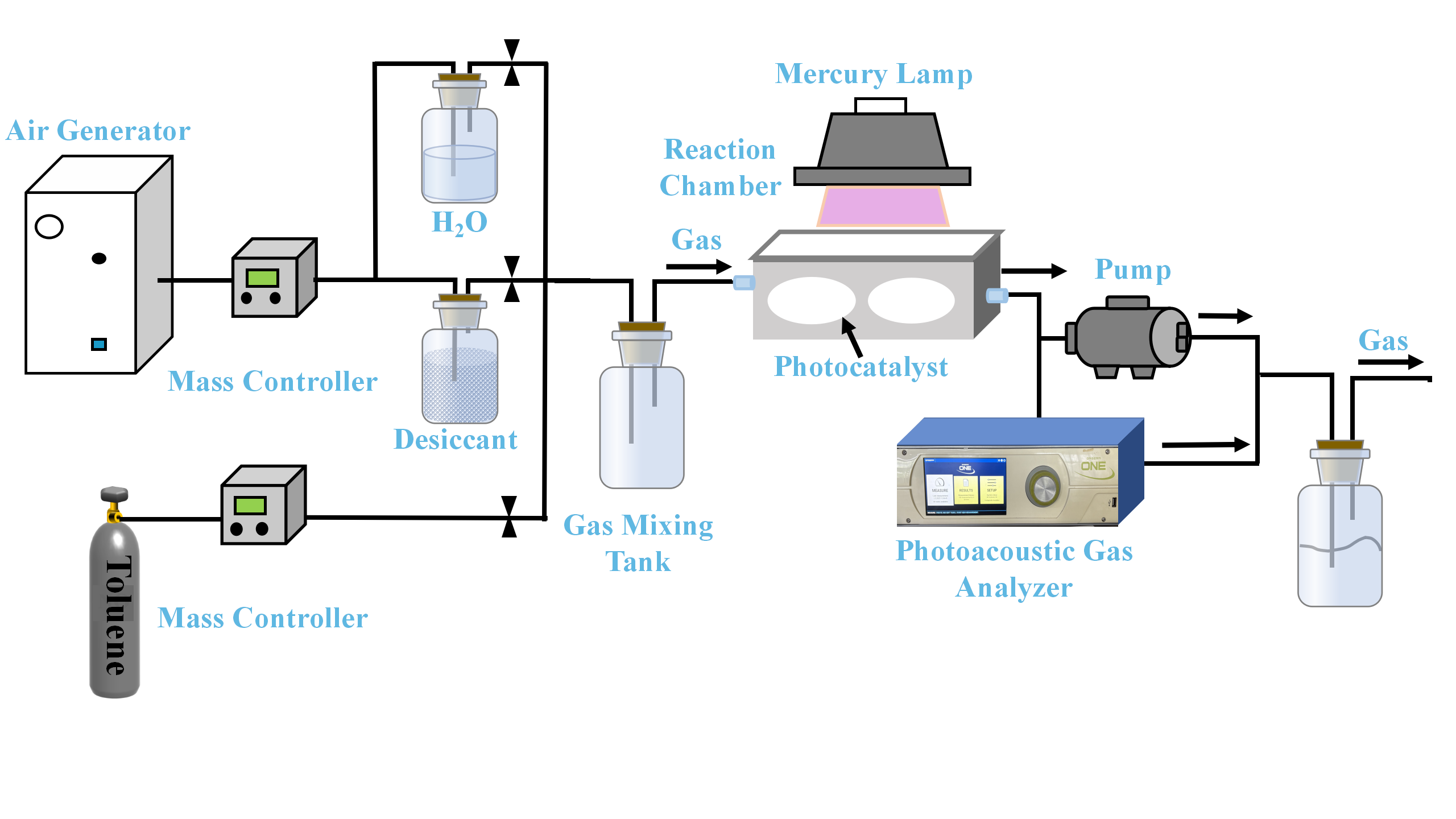


**Scheme S1.** Schematic diagram of toluene photo-oxidation under continuous gas flow.

**
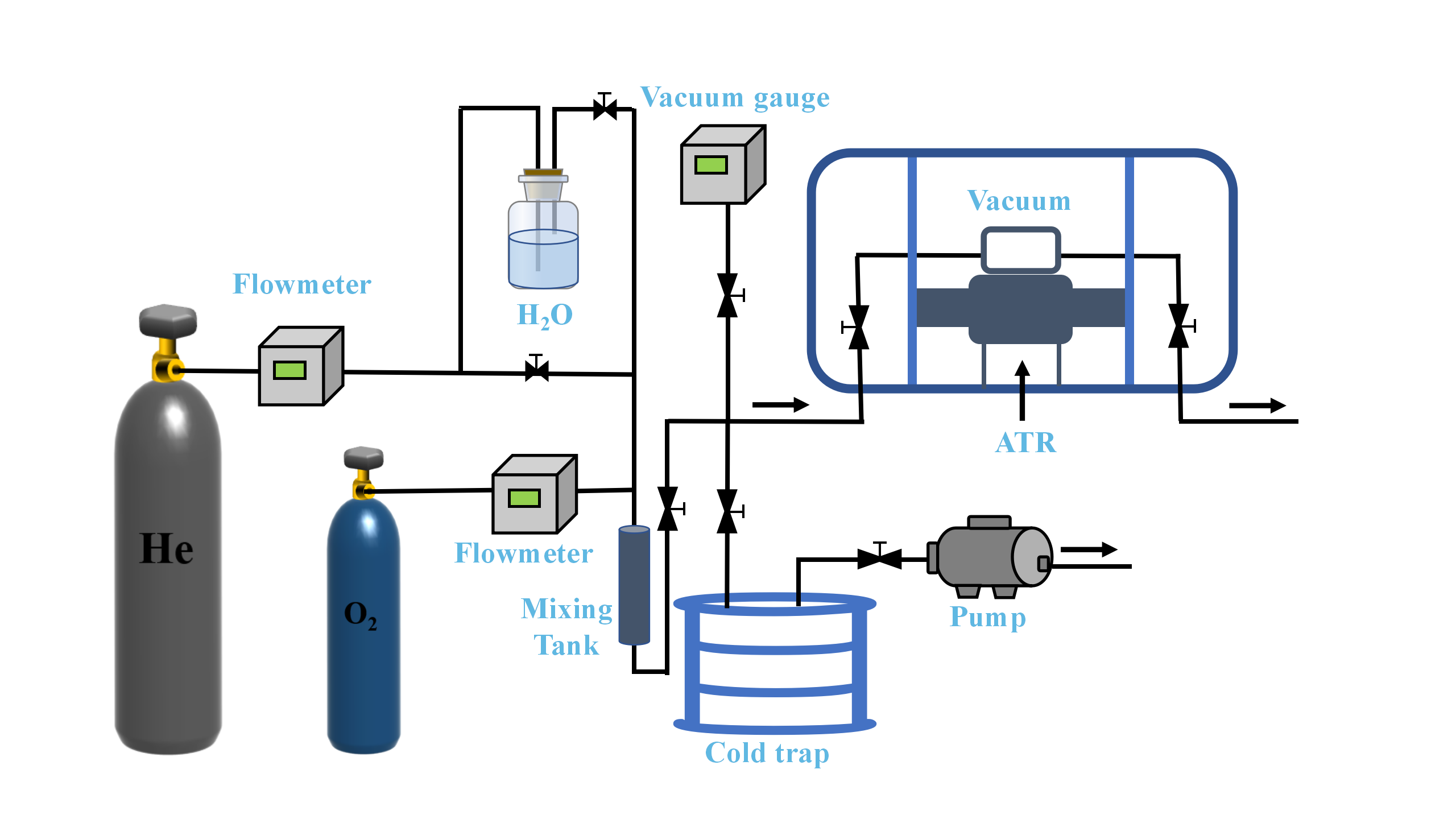
**

**Scheme S2.** Illustration of the in-situ ATR-FTIR spectroscopy system.**
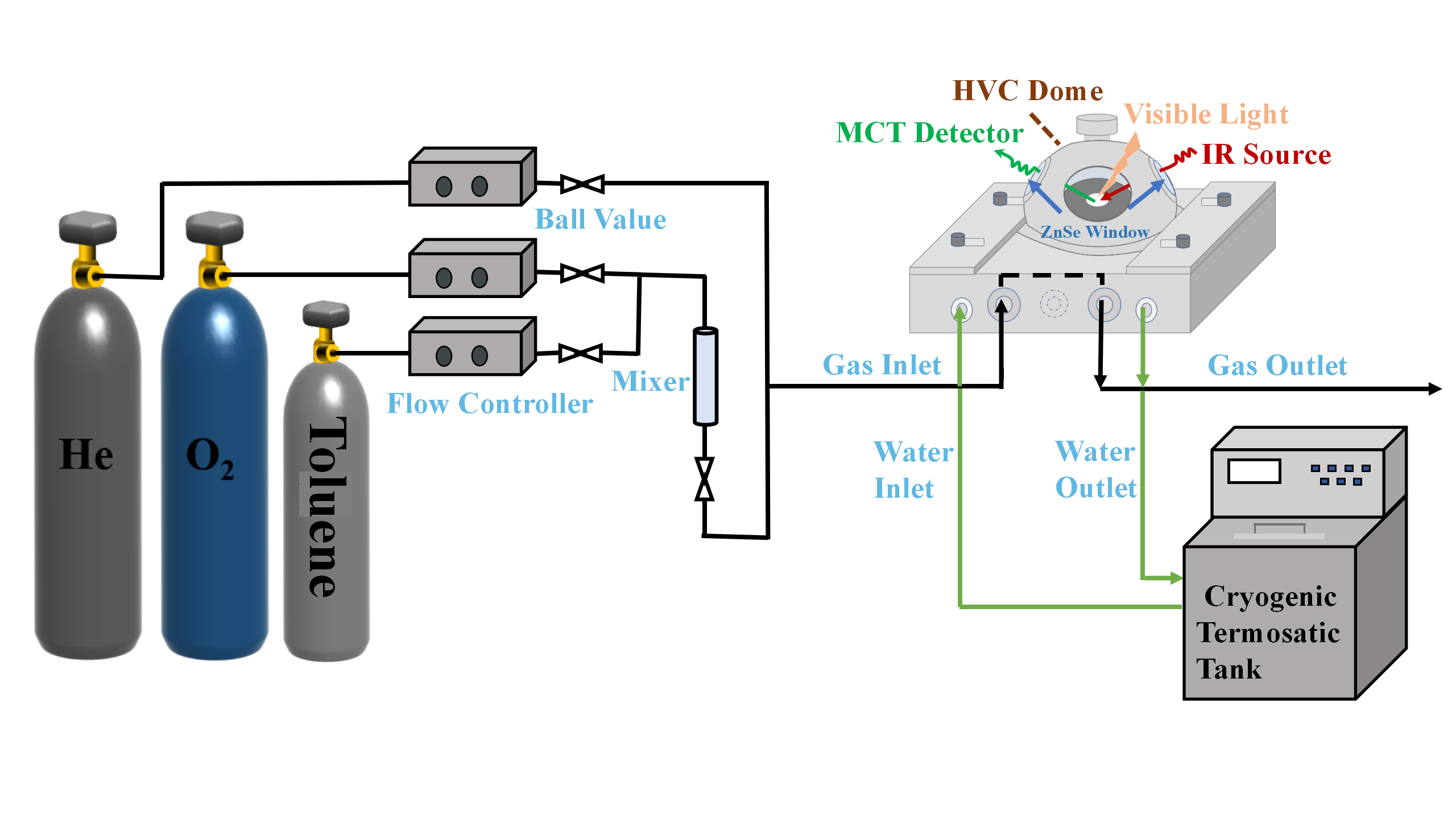
**

**Scheme S3.** Illustration of the in-situ DRIFTS spectroscopy system.

# Figures

**
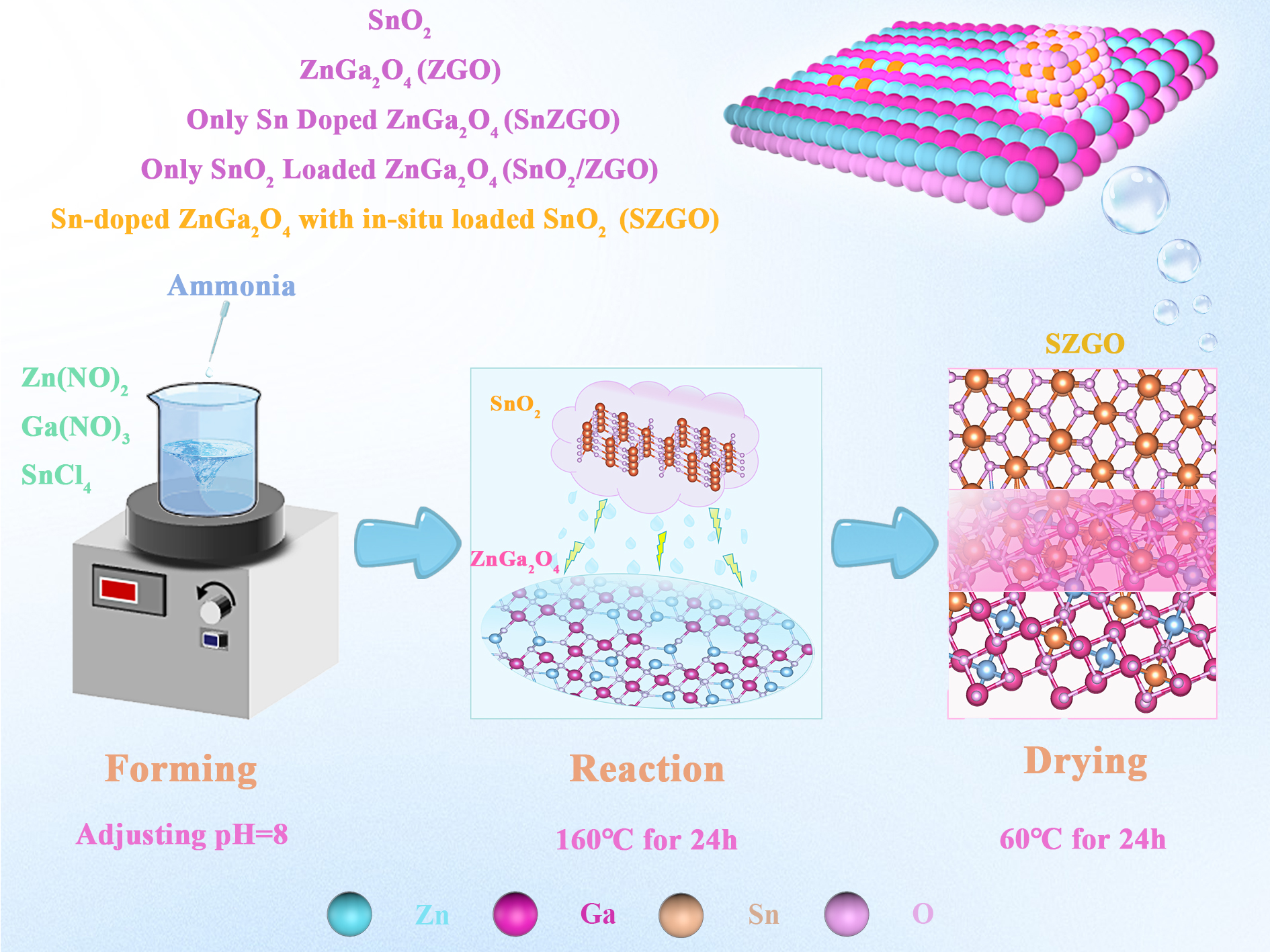
**

**Figure S1.** Schematic of the synthesis strategy for the prepared samples.


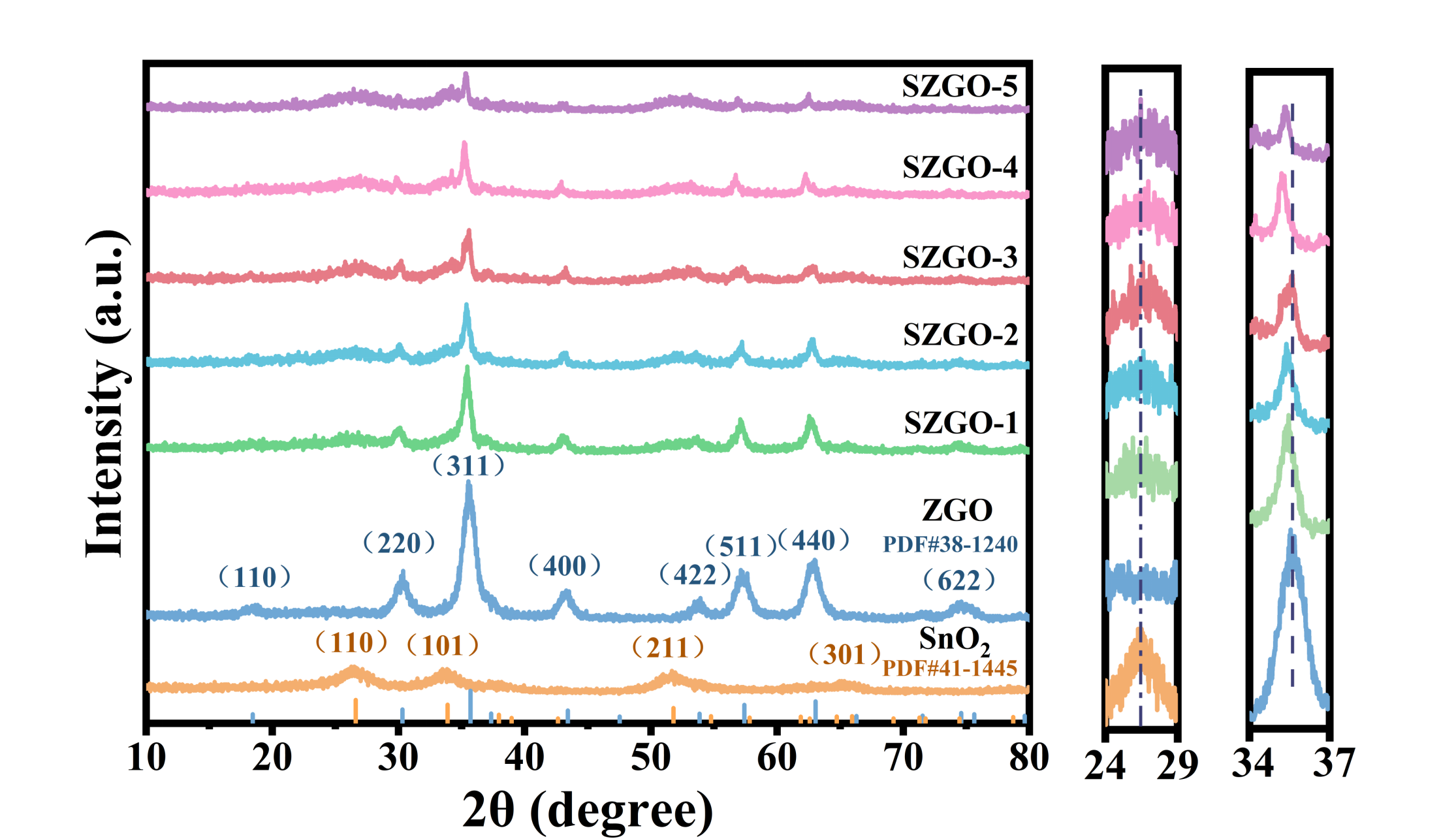


**Figure S2.** XRD patterns of the as-prepared samples with partial magnification in the ranges of 24°-29° and 34°-37°.

**
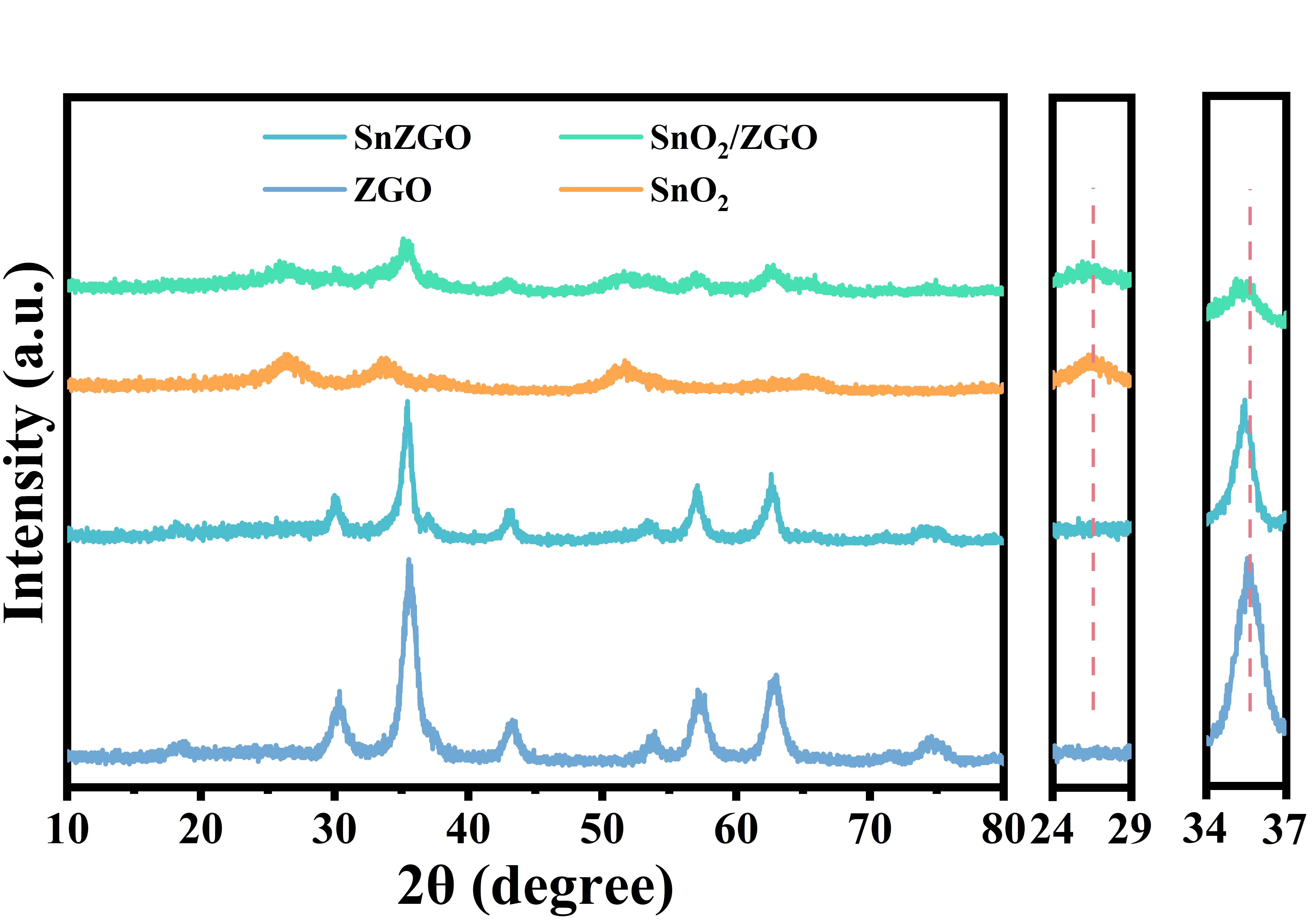
**

**Figure S3.** XRD patterns of SnZGO and SnO_2_/ZGO with enlarged views in the regions of 24°-29° and 34°-37°.


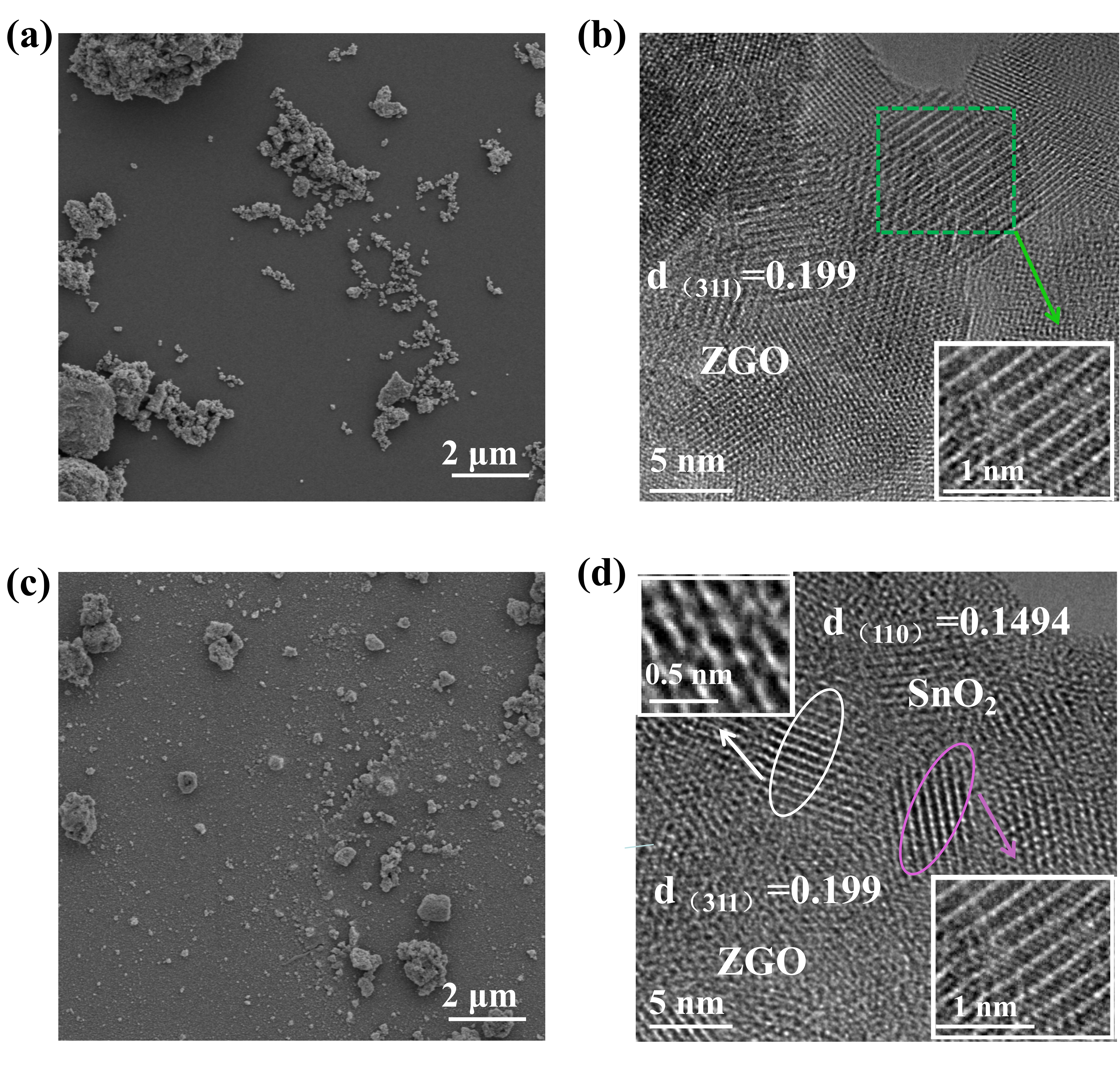


**Figure S4.** SEM images of a) ZGO and c) SZGO. HRTEM images of b) ZGO and d) SZGO.

**
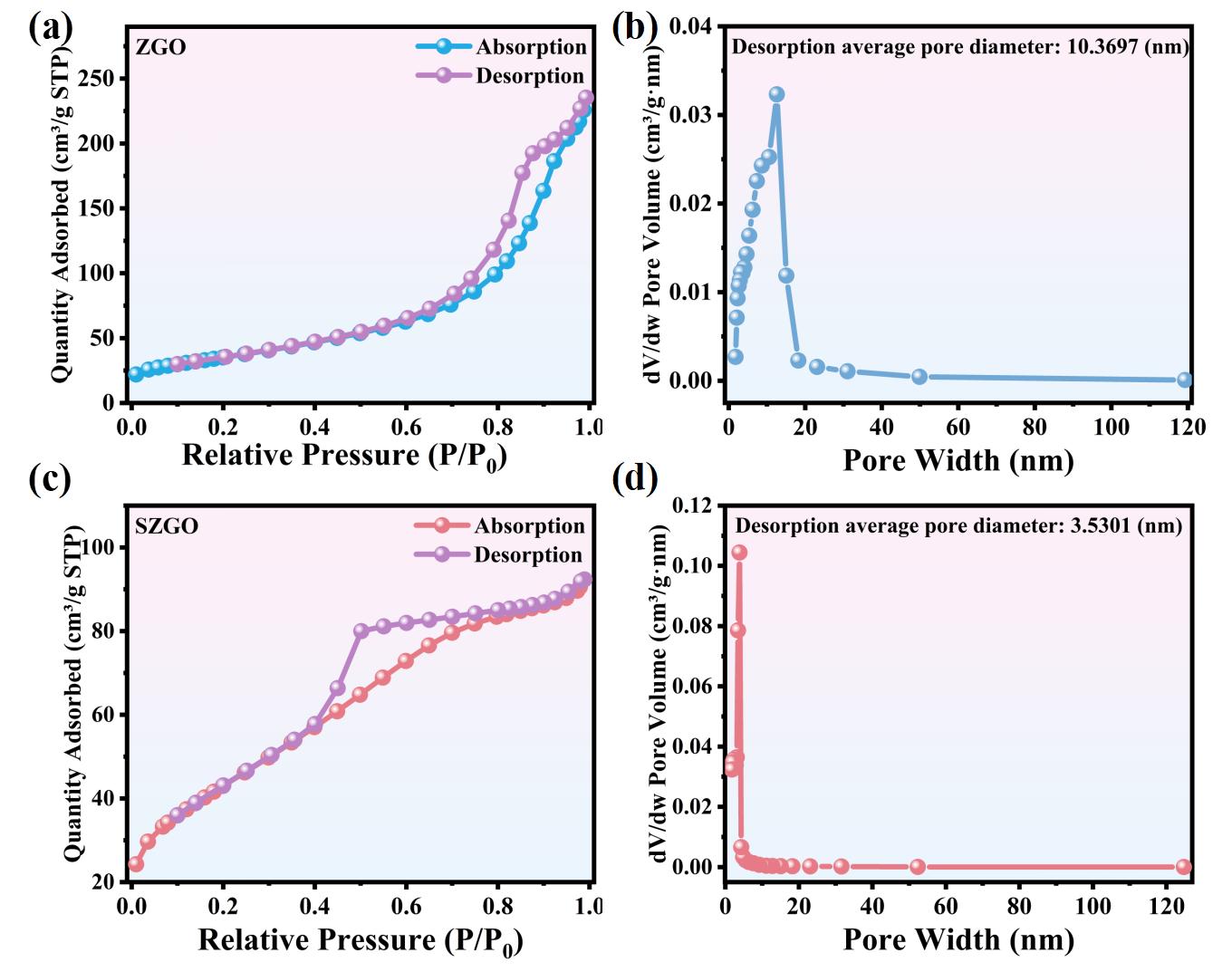
**

**Figure S5.** N_2_ adsorption–desorption isotherms of a) ZGO and c) SZGO, and the corresponding desorption average pore diameter distributions of b) ZGO and d) SZGO.

**
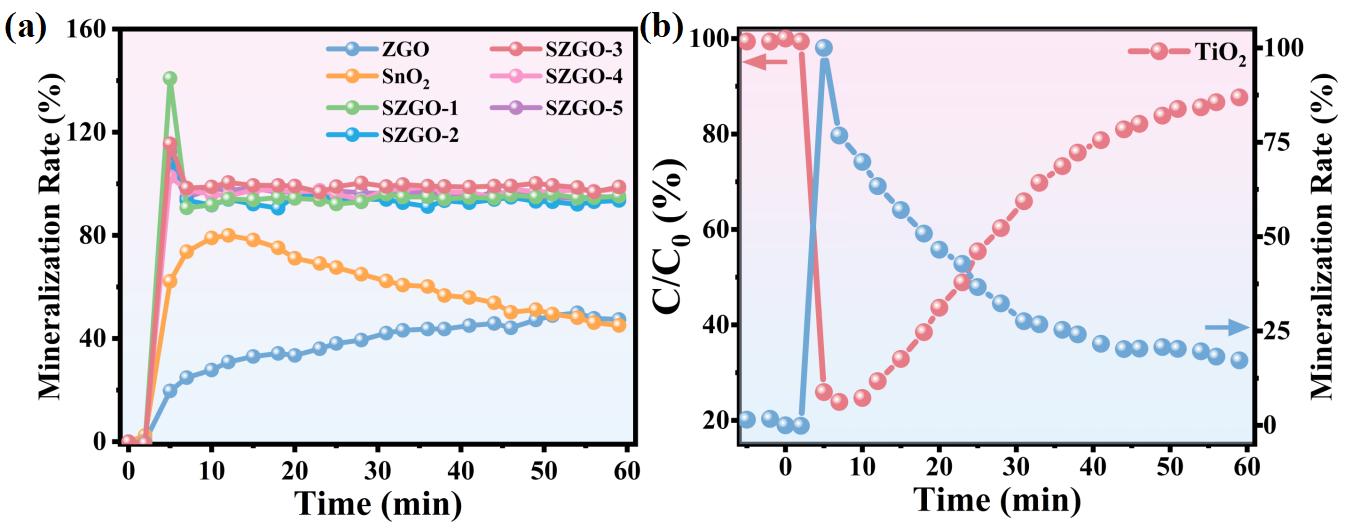
**

**Figure S6.** a) Mineralization rate of the as-prepared samples. b) Toluene degradation efficiency and mineralization rate of commercial TiO_2_ under UV illumination.


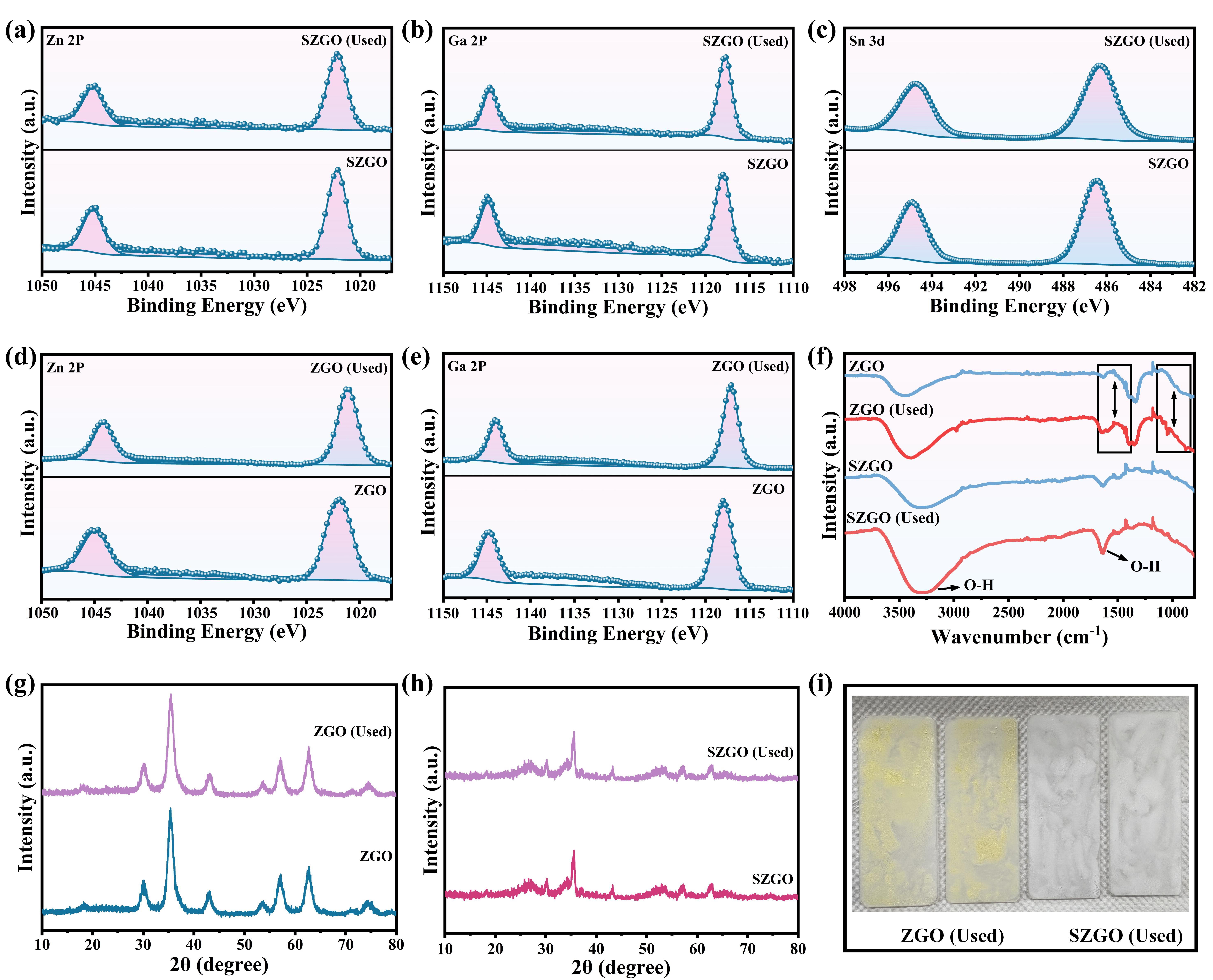


**Figure S7.** a-e) XPS spectra of SZGO and ZGO before and after the test. f) FTIR spectra of SZGO and ZGO before and after the test. XRD pattern of g) ZGO and h) SZGO before and after the test. i) images showing the samples before and after the test.


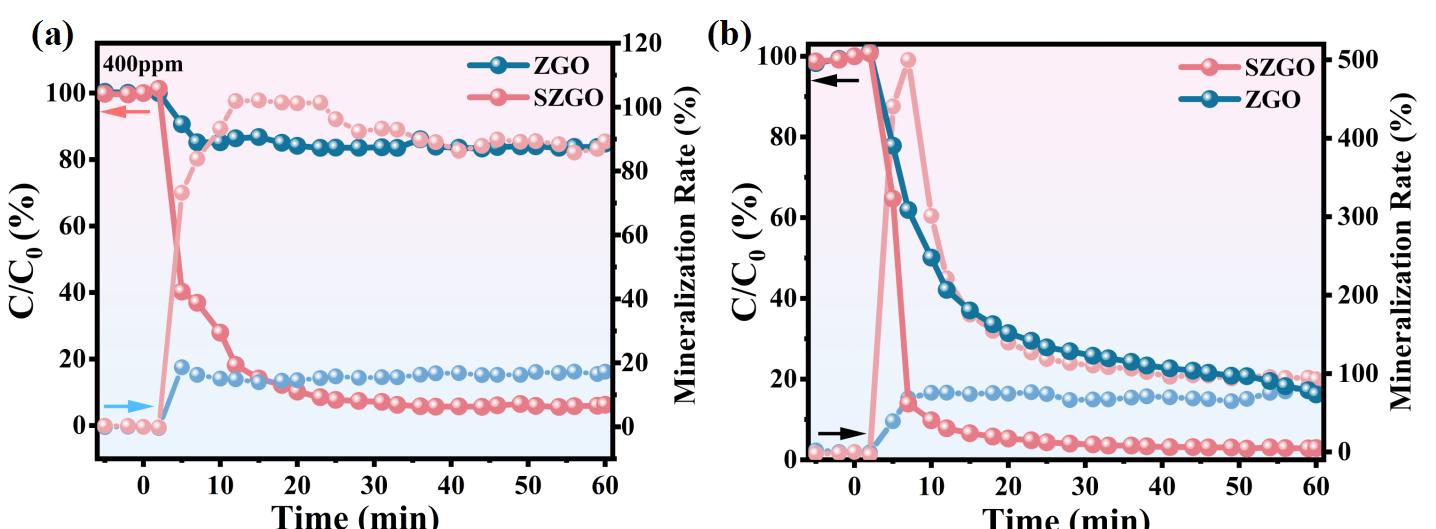


**Figure S8.** Degradation efficiency and mineralization rate of a) acetone and b) formaldehyde under UV light irradiation.


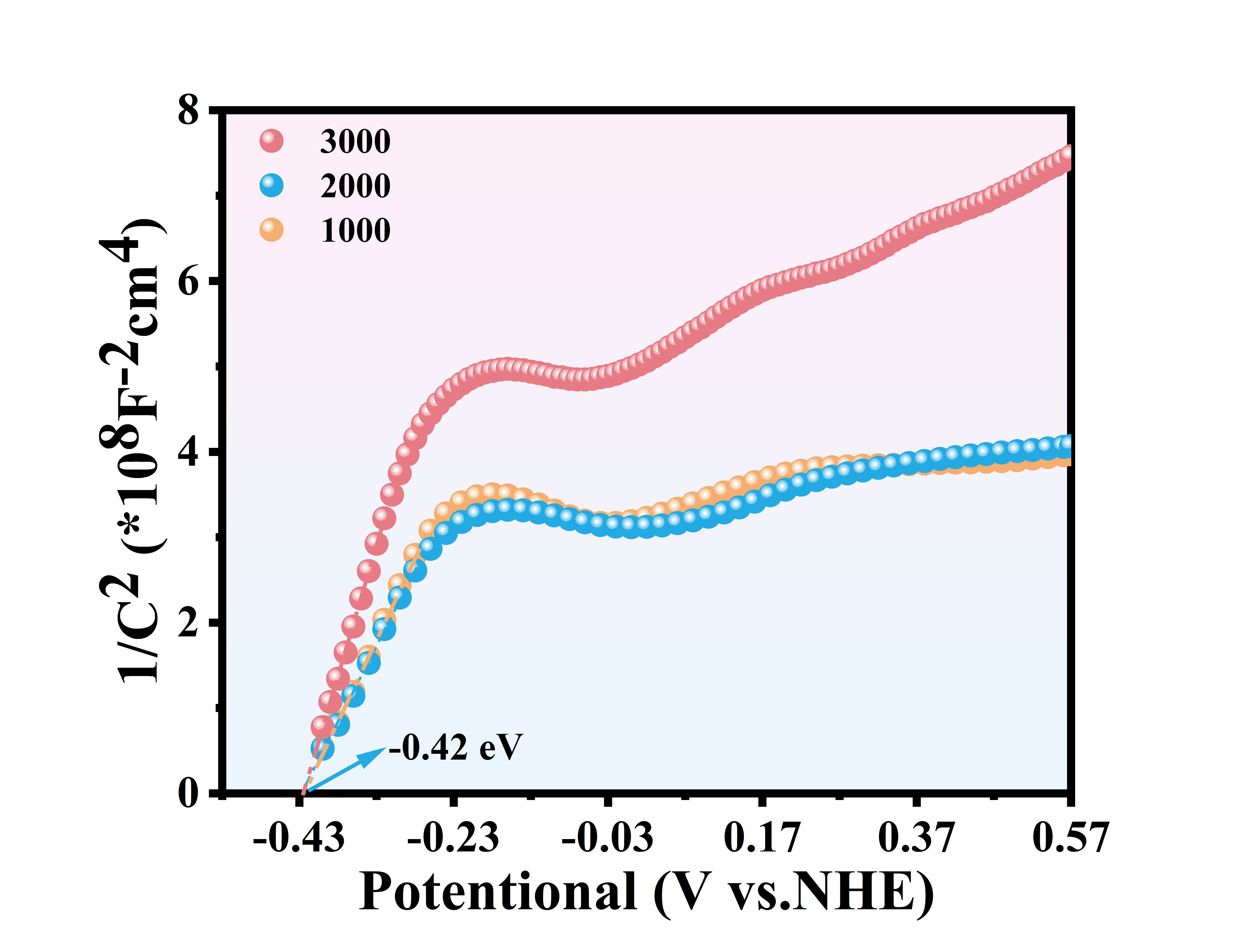


**Figure S9.** Mott Schottky spectra of SnO_2_.


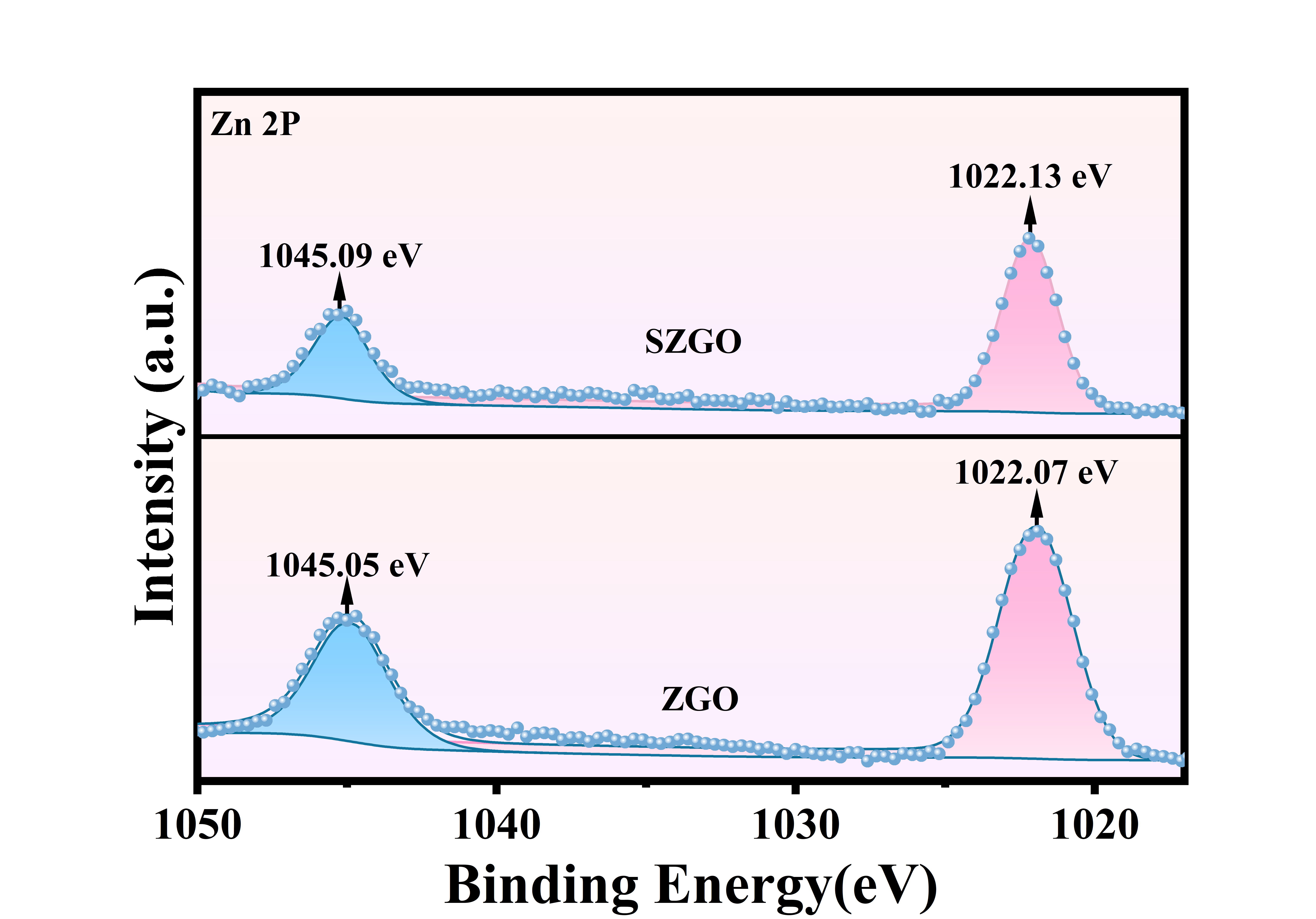


**Figure S10.** Zn 2p XPS spectra of ZGO and SZGO.


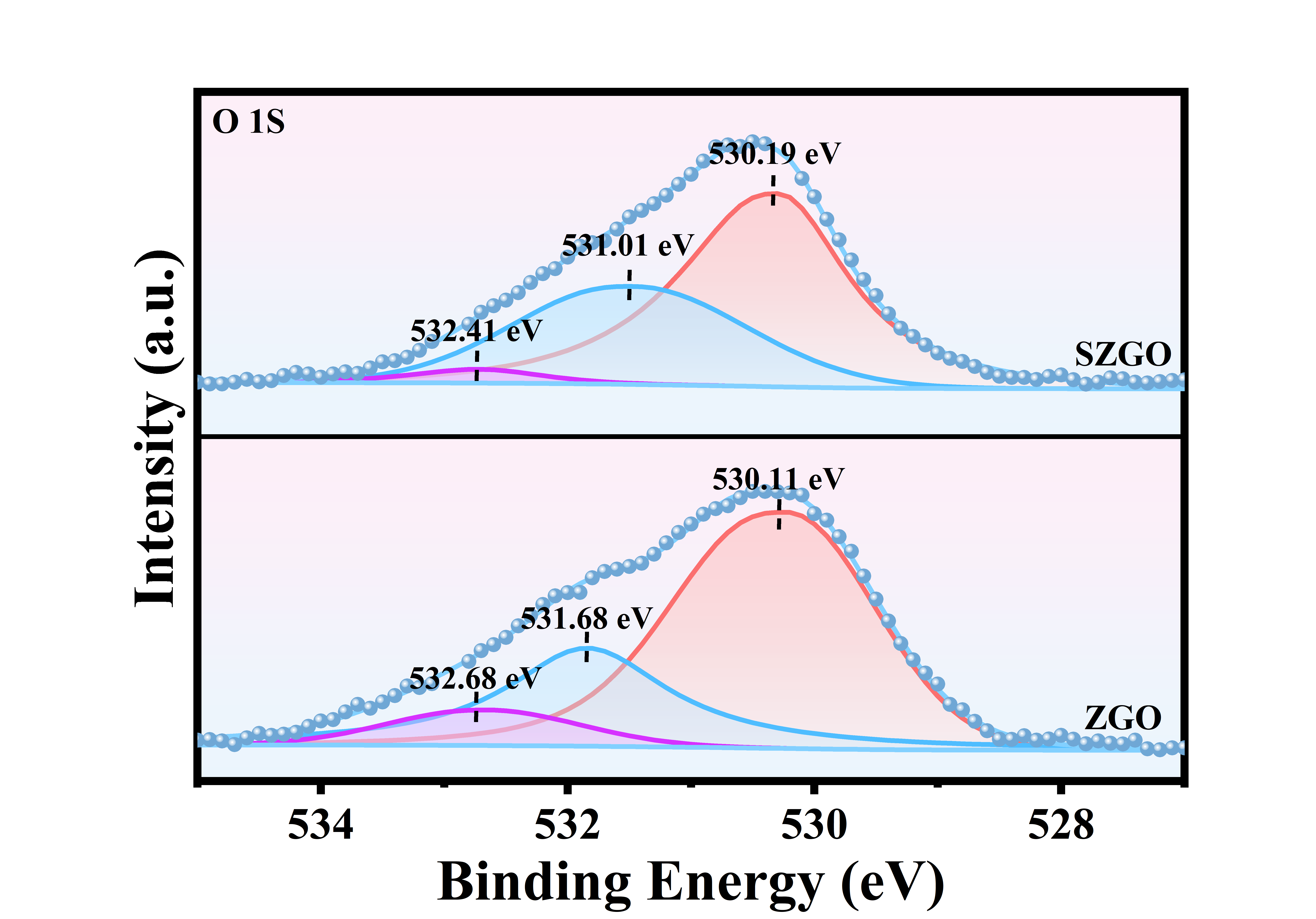
.

**Figure S11.** O 1s XPS spectra of ZGO and SZGO.


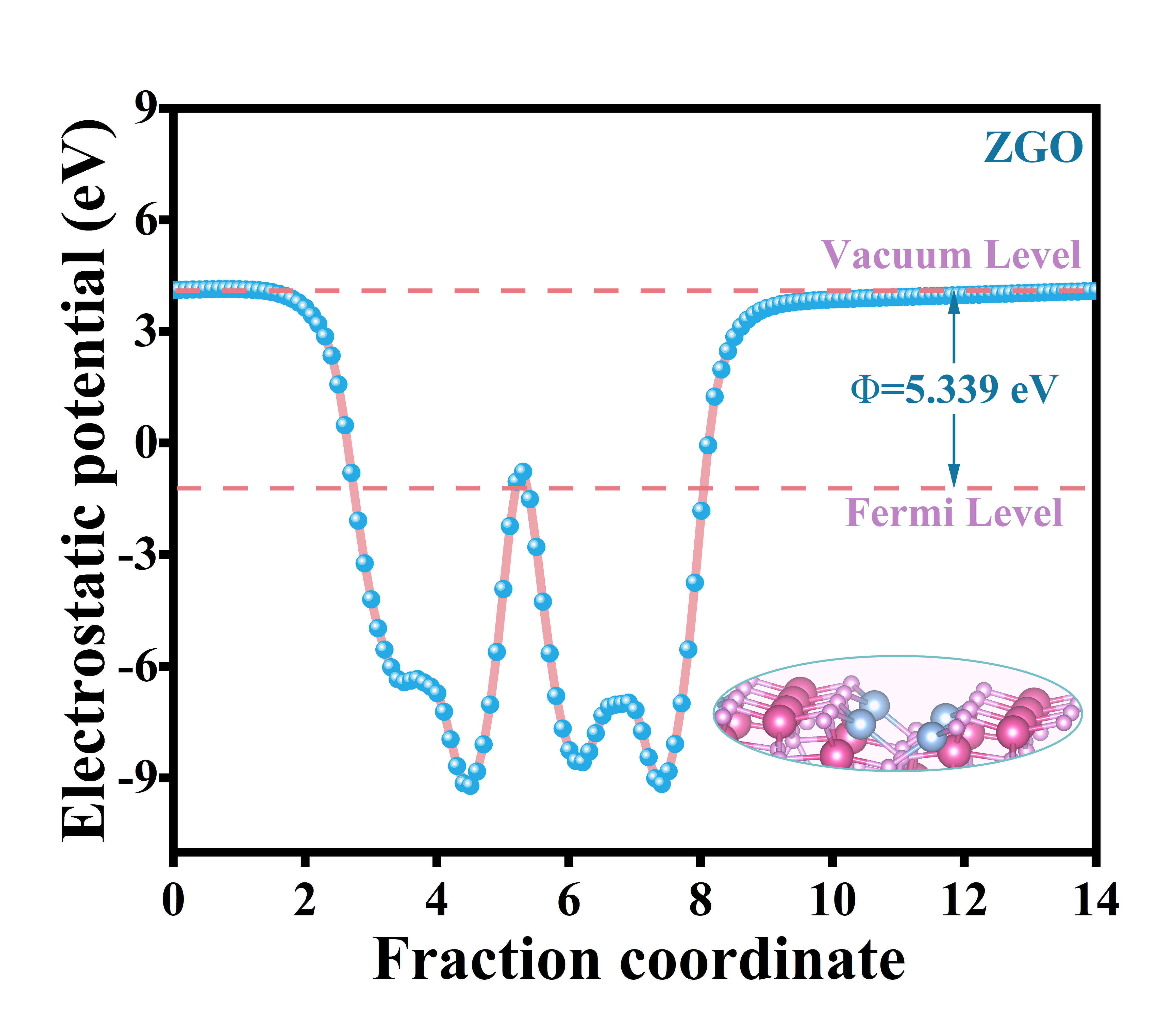


**Figure S12.** Work function and the relative Fermi level of ZGO.


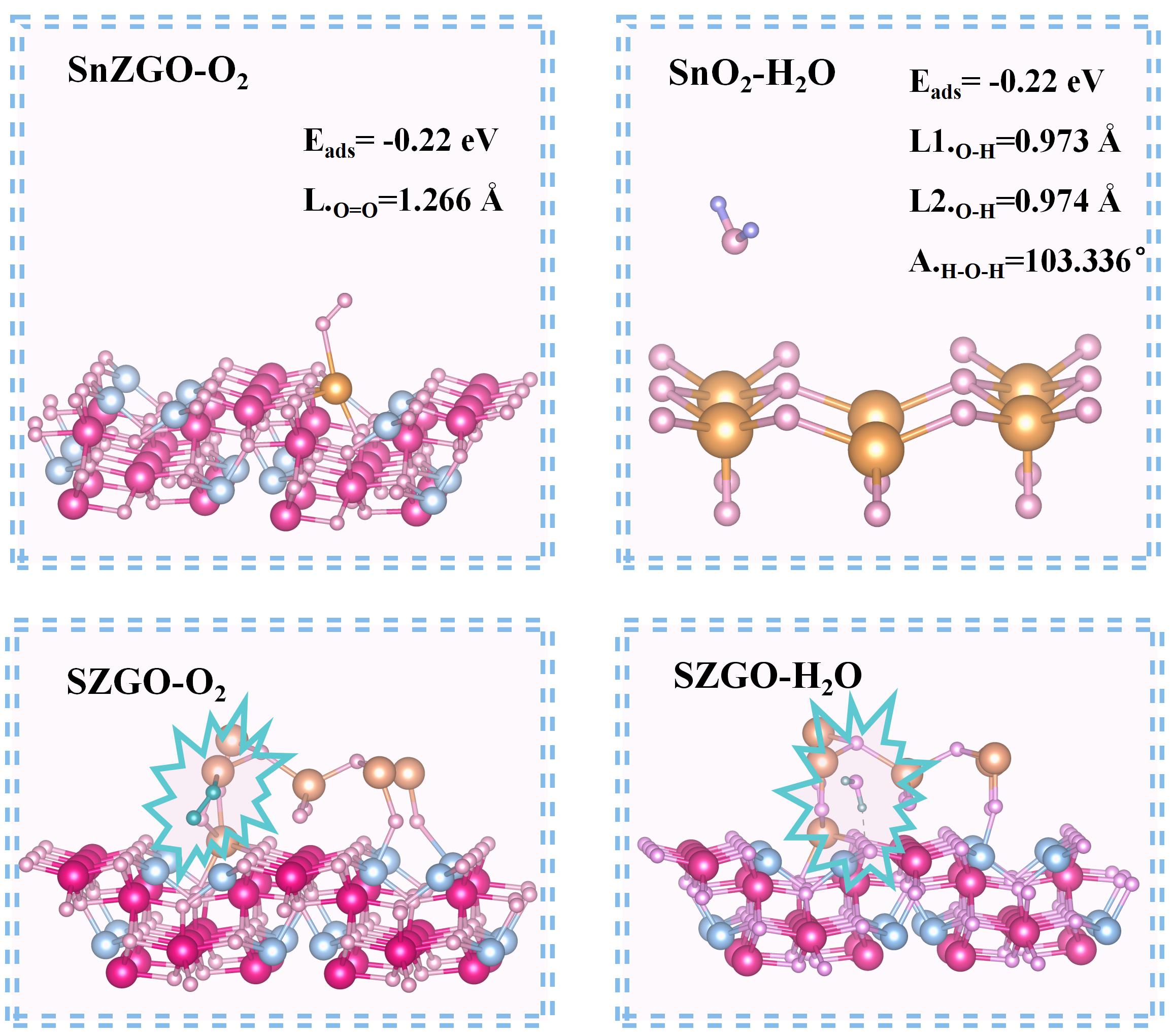


**Figure S13.** Adsorption models of O_2_ molecules on a) SnZGO and c) SZGO. Adsorption models of H_2_O molecules on b) SnO_2_ and d) SZGO.


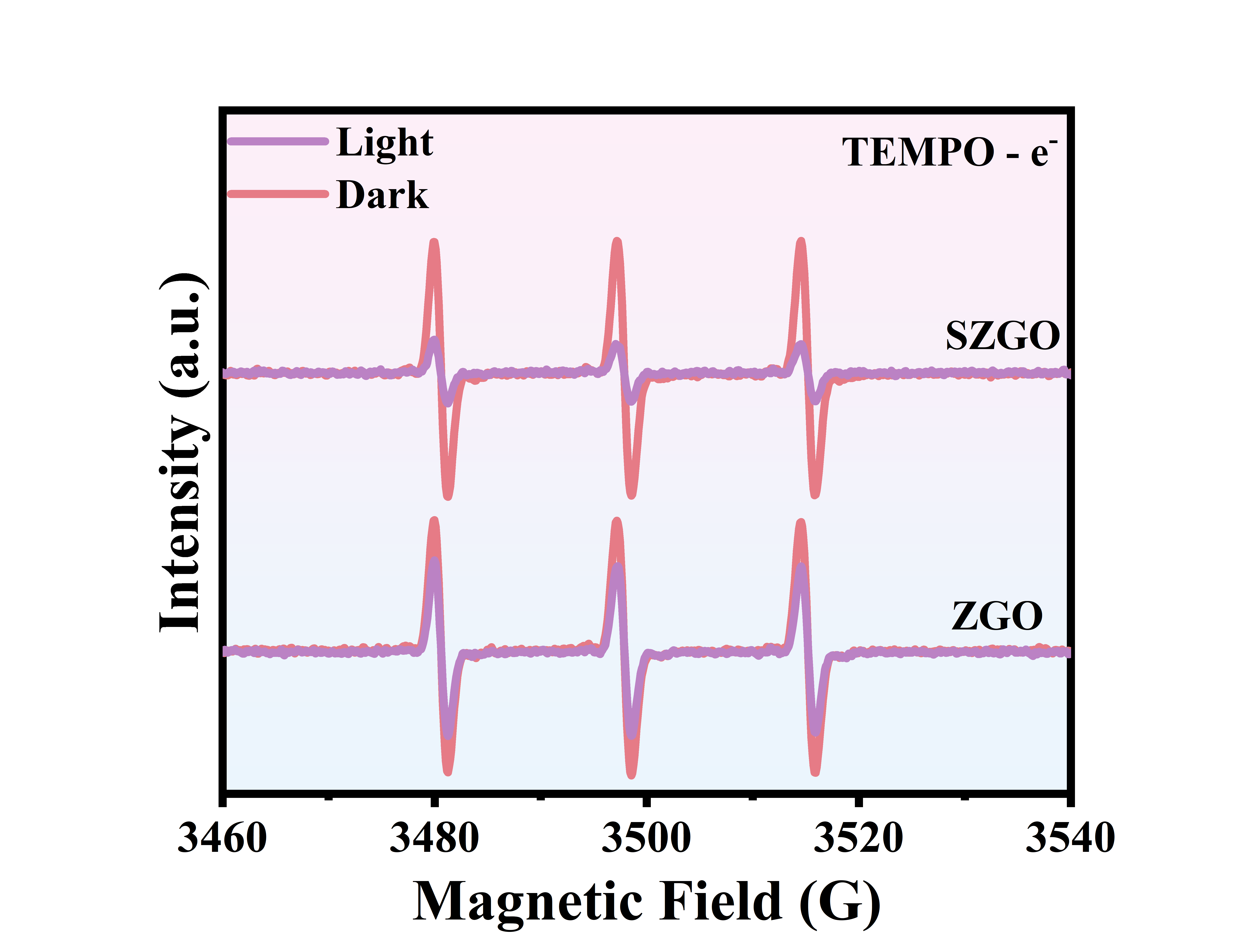


**Figure S14.** EPR spectra of TEMPO- e^-^.


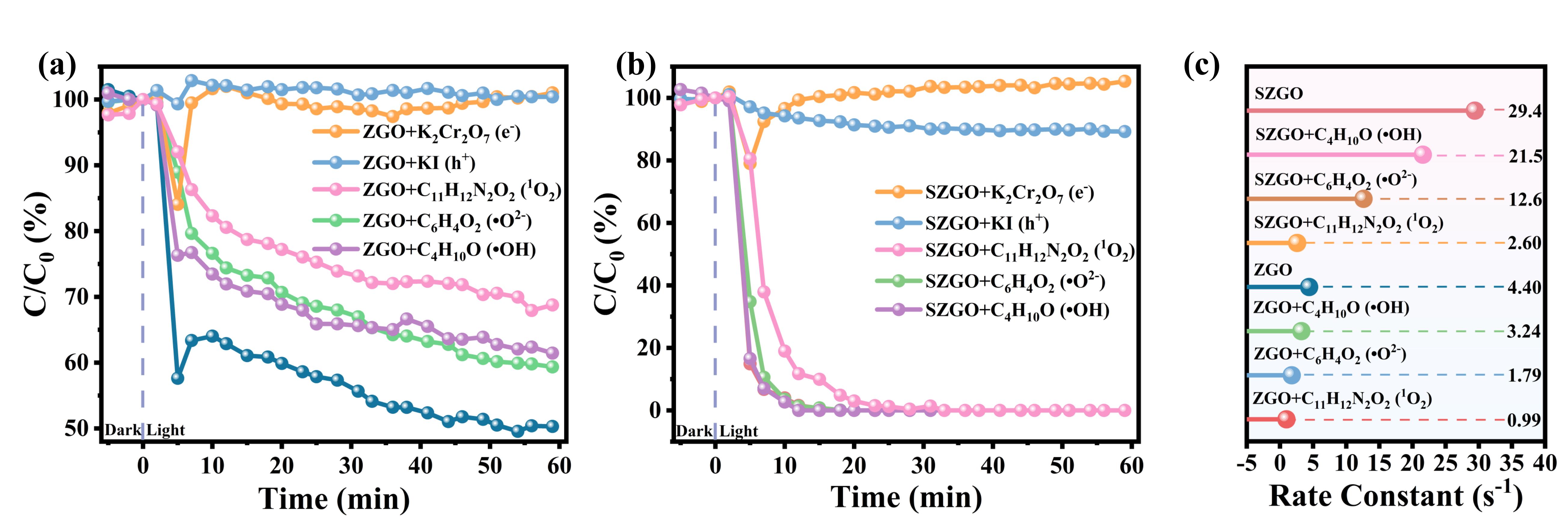


**Figure S15.** Comparison of free radical quenching behavior for a) ZGO and b) SZGO using scavenger experiments, and c) the corresponding first-order rate constant.


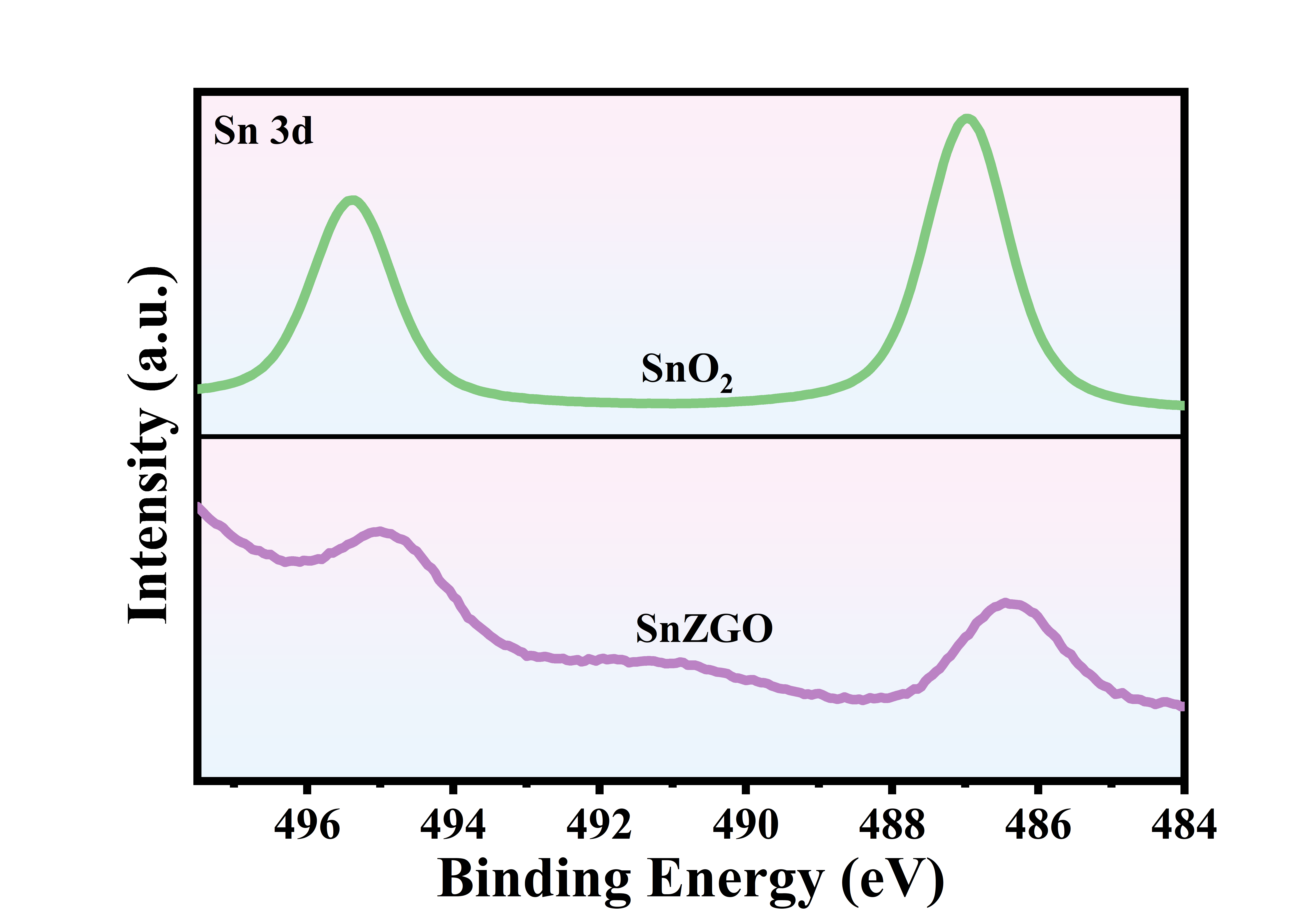


**Figure S16.** Sn 3d XPS spectra of SnZGO and SnO_2_.


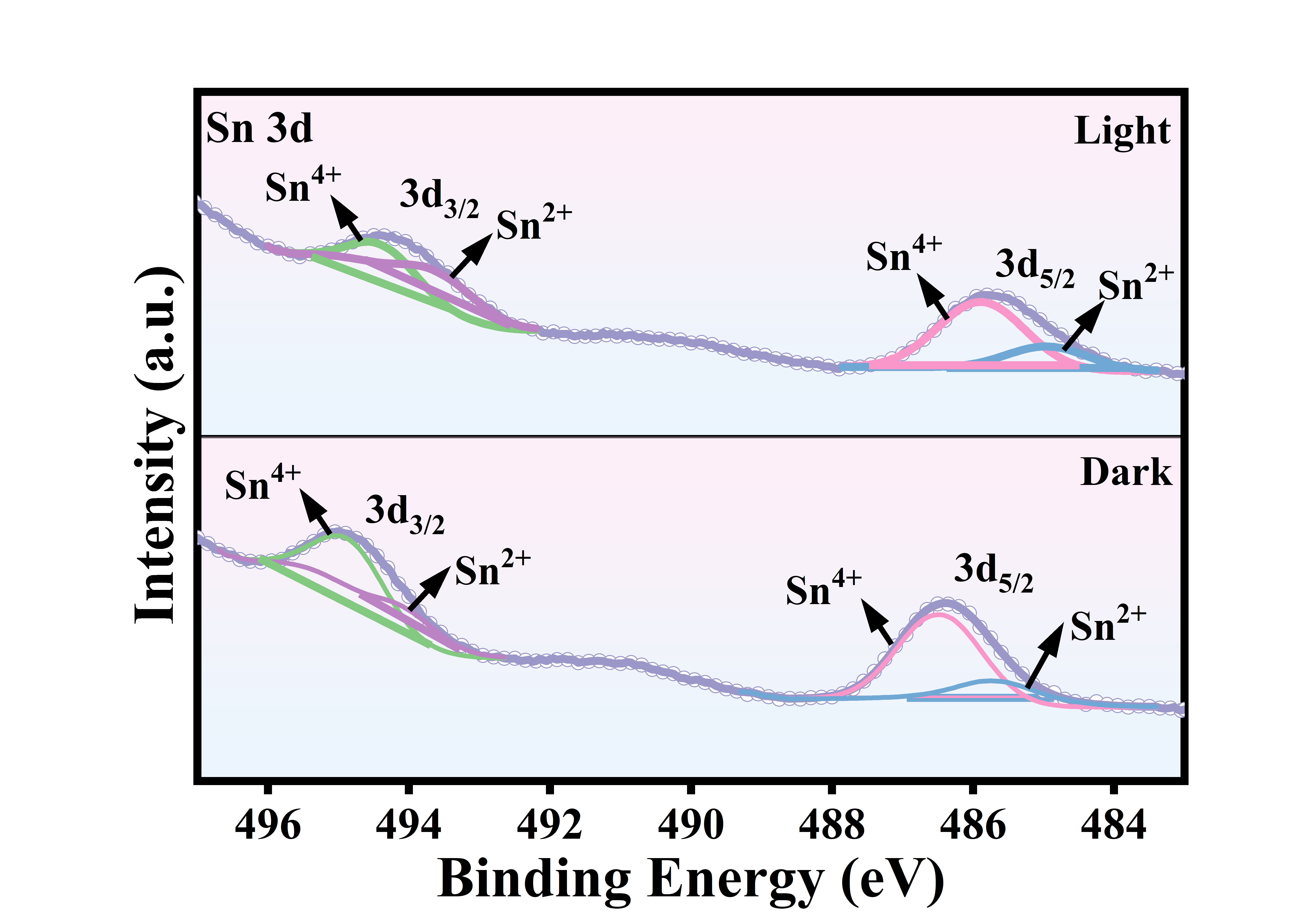


**Figure S17.** Quasi in-situ high-resolution XPS spectra of Sn 3d for SnZGO under dark and illumination conditions.


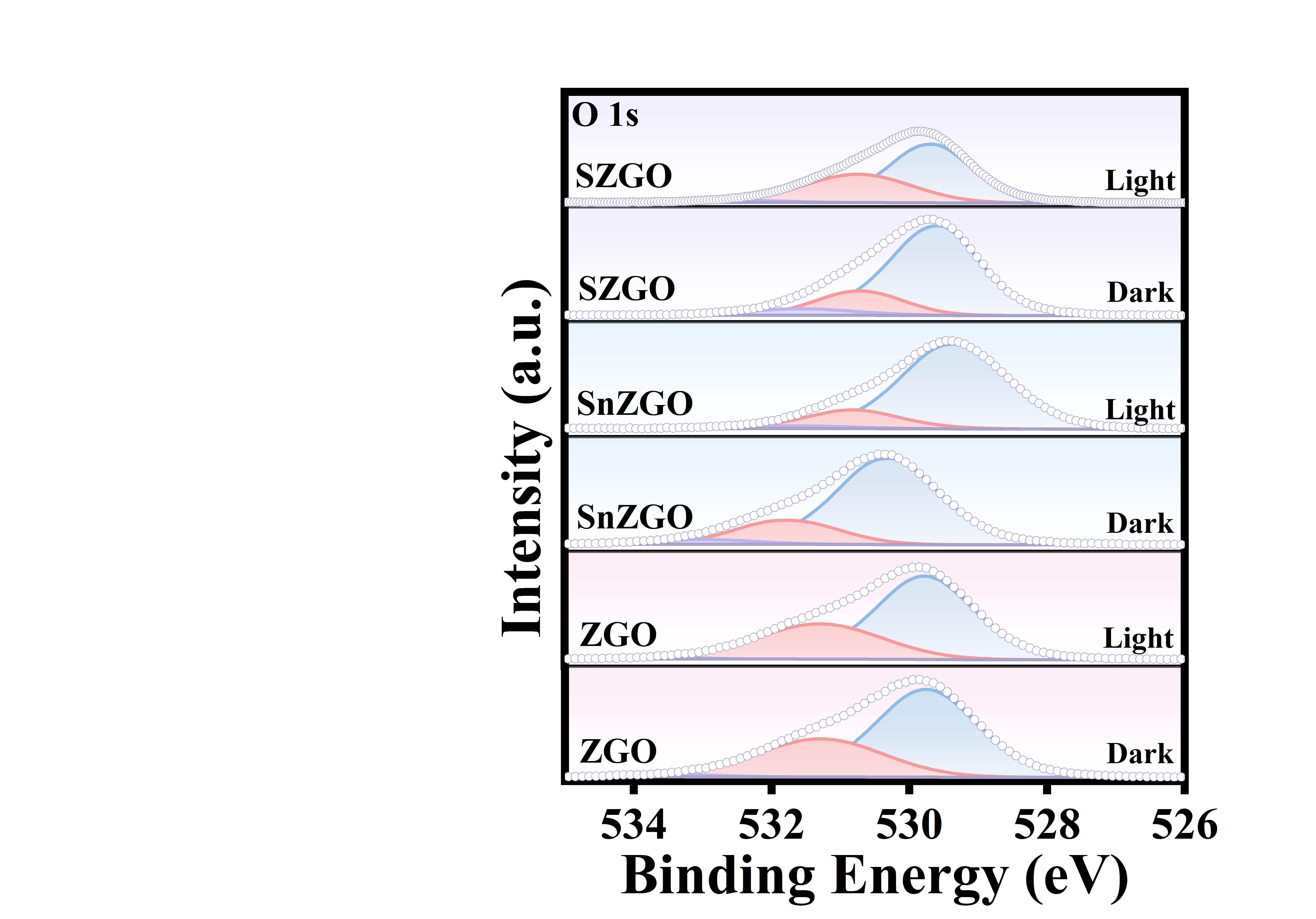


**Figure S18.** Quasi in-situ high-resolution XPS spectra of O 1s for ZGO, SnZGO and SZGO under dark and illumination conditions;

**
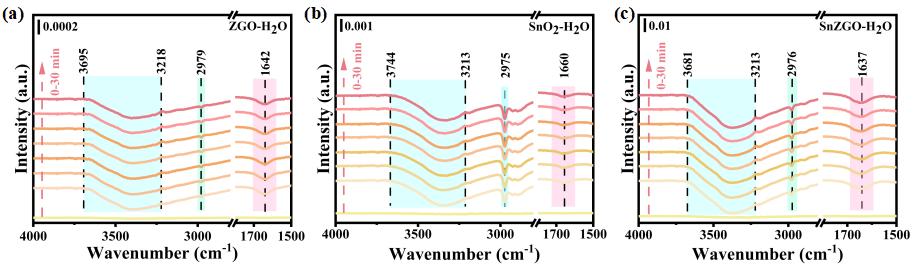
**

**Figure S19.** In-situ ATR-FTIR spectra of H_2_O adsorption on a) ZGO, b) SnO_2_ and c) SnZGO.


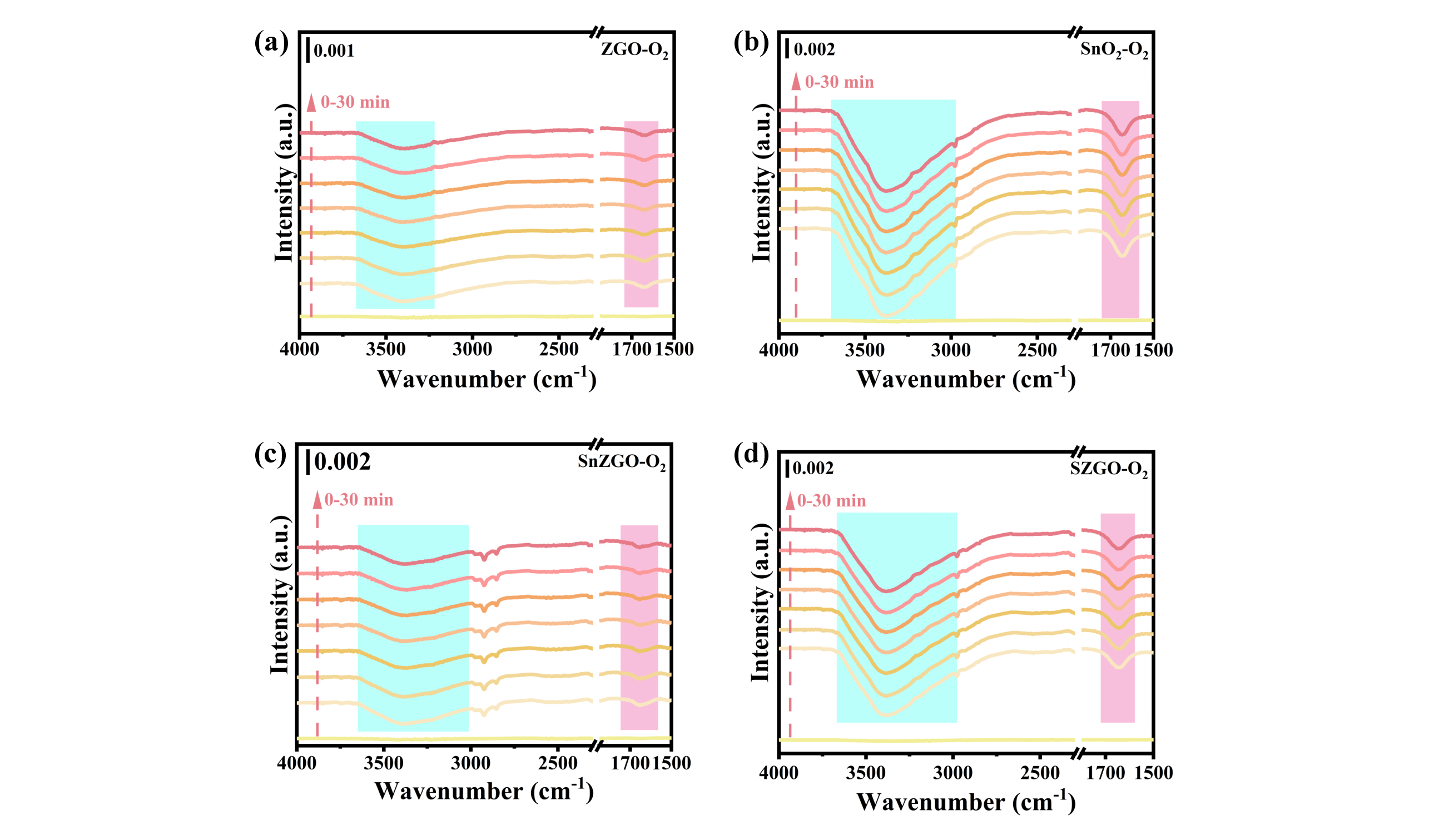


**Figure S20.** In-situ ATR-FTIR spectra of O_2_ adsorption on a) ZGO, b) SnO_2_, c) SnZGO and d) SZGO.

**
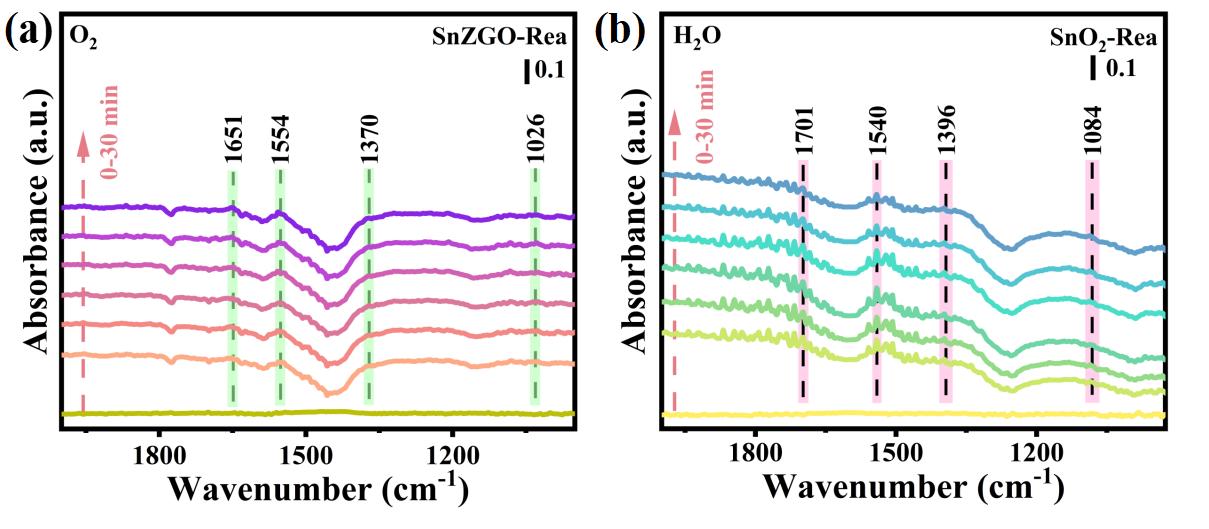
**

**Figure S21.** In-situ DRIFTS spectra of toluene photo-oxidation on the a) SnZGO under O_2_-rich conditions and b) SnO_2_ under H_2_O-rich conditions.

# References

1. S. Liang, Y. Shu, K. Li, J. Ji, H. Huang, J. Deng, D.Y.C. Leung, M. Wu, Y. Zhang, *J. Hazard. Mater.*, **2020**, *399*, 122967.
2. J. Zhang, Z. Guo, Z. Yang, J. Wang, J. Xie, M. Fu, Y. Hu, *ChemCatChem*, **2020**, *13*, 581.
3. P. Chen, L. Chen, X. Dong, H. Wang, J. Li, Y. Zhou, X. Chao, Y. Zhang, F. Dong, *ACS EST Engg.* **2020**, *1*, 501.
4. H. Wu, J. Wang, R. Chen, C. Yuan, J. Zhang, Y. Zhang, J. Sheng, F. Dong, *Chin. J. Catal.* **2021**, *42*, 1195.
5. Y. Liu, S. Chen, K. Li, J. Wang, P. Chen, H. Wang, J. Li, F. Dong, *J. Colloid Interface Sci.* **2022**, *606*, 1435.
6. L. Chen, K. Li, Y. Yang, T. Xue, H. Wang, B. Lei, J. Sheng, F. Dong, Y. Sun, *J. Hazard. Mater.* **2023**, *444*, 130436.
7. [B. Lei](https://onlinelibrary.wiley.com/authored-by/Lei/Ben), [W. Cui](https://onlinelibrary.wiley.com/authored-by/Cui/Wen), [P. Chen](https://onlinelibrary.wiley.com/authored-by/Chen/Peng), [R. Chen](https://onlinelibrary.wiley.com/authored-by/Chen/Ruimin), [Y. Sun](https://onlinelibrary.wiley.com/authored-by/Sun/Yanjuan), [K. H. Kim](https://onlinelibrary.wiley.com/authored-by/Kim/Ki%E2%80%90Hyun), [F. Dong](https://onlinelibrary.wiley.com/authored-by/Dong/Fan), *Energy Environ. Mater.* **2023**, *6*, e12291.
8. B. Liu, B. Zhang, B. Liu, Z. Hu, W. Dai, J. Zhang, F. Feng, B. Lan, T. Zhang, H. Huang, *Environ. Sci. Technol.* **2024**, *58*, 4404.
9. Y. Li, B. Chen, L. Liu, B. Zhu, D. Zhang, *Angew. Chem. Int. Ed*. **2024**, *63*, 202319432.
10. J. Wang, C. Yang, D. Ye, Y. Hu, *Appl. Catal. B-Enviro*, **2025**, *361*, 124635.
11. H. Ma, X. Hu, X. Wang, W. Xu, Y. Shen, R. Fang, Y. Li, Y. Liu, F. Dong, *Appl*. *Catal. B-Enviro*. **2025**, *361*, 124638.
12. H. Sheng, H. Zhang, W. Song, H. Ji, W. Ma, C. Chen, J. Zhao, *Angew. Chem., Int. Ed*. **2015**, *54*, 5905.
13. S. Zhao, F. Hu, J. Li, *ACS Catal.* **2016**, *6*, 3433.
14. H. Wu, J. Wang, R. Chen, C. Yuan, J. Zhang, Y. Zhang, J. Sheng, F. Dong, *Chinese J. Catal.* **2021**, *42*, 1195.
15. H. Lin, J. Long, Q. Gu, W. Zhang, R. Ruan, Z. Li, X. Wang, Phys. *Chem. Chem. Phys.* **2012**, *14*, 9468.
16. A.H. Mamaghani, F. Haghighat, C.-S. Lee, *Chem. Eng. J.* **2018,** *337*, 60.
17. X. Zhou, X. Wang, T. Tan, H. Ma, H. Tang, X. Luo, F. Dong, Y. Yang, *Chem. Eng. J.* **2023**, *470*, 143933.
18. X. Yang, X. Yu, M. Lin, X. Ma, M. Ge, *Catal. Today*, **2019**, *327*, 254.
19. J. Liu, Y. Li, J. Ke, S. Wang, L. Wang, H. Xiao, *Appl. Catal. B Environ*. **2018**, *224*, 705.
20. J. Li, K. Li, B. Lei, M. Ran, Y. Sun, Y. Zhang, [K. Kim](https://www.sciencedirect.com/author/56962774000/ki-hyun-kim), [F. Dong,](https://www.sciencedirect.com/author/35315446400/fan-dong) *[Chem. Eng. J.](https://www.sciencedirect.com/author/35315446400/fan-dong)* **[2021](https://www.sciencedirect.com/author/35315446400/fan-dong)***[, 413,](https://www.sciencedirect.com/author/35315446400/fan-dong)* [127389](https://www.sciencedirect.com/author/35315446400/fan-dong)*[.](https://www.sciencedirect.com/author/35315446400/fan-dong)*
21. H. Wang, Q. Ren, L. Xiao, L. Chen, Y. He, L. Yang, Y. Sun, F. Dong, *J. Hazard. Mater.* **2022**, *437*, 129329.
22. W. He, J. Li, X. Hou, P. Chen, H. Wang, X. Dong, F. Dong, Y. Sun, *Chem. Eng. J.* **2022**, *427*, 131764.
23. J. Liu, Y. Li, Z. Li, J. Ke, H. Xiao, Y. Hou, *Catal. Today*, **2018**, *314*, 2.
24. J. Zhang, B. Shen, Z. Hu, M. Zhen, S. Guo, F. Dong, *Appl. Catal. B-Enviro*. **2021**, *296*, 120376.
25. Y. Liu, S. Chen, K. Li, J. Wang, P. Chen, H. Wang, J. Li, F. Dong, *J. Colloid and Interf*. *Sci.* **2022**, *606*, 1435.
26. C. Chen, Y. Peng, J. Chen, C. Wang, H. Yin, H. Wang, C. You, J. Li, *Environ. Sci. Technol.* **2020**, *54*, 14465.
27. J. Wang, J. Li, W. Yang, Y. Liu, H. Wang, Q Geng, [F. Dong,](https://www.sciencedirect.com/author/35315446400/fan-dong) *Appl. Catal. B Environ.* **2021**, *297*, 120489.
28. [B. Chen](https://advanced.onlinelibrary.wiley.com/authored-by/Chen/Bangfu), [Y. Duan](https://advanced.onlinelibrary.wiley.com/authored-by/Duan/Youyu), [J. Tan](https://advanced.onlinelibrary.wiley.com/authored-by/Tan/Jing), [Y. Li](https://advanced.onlinelibrary.wiley.com/authored-by/Li/Yuhan), [S. Li](https://advanced.onlinelibrary.wiley.com/authored-by/Li/Shuangjun), [D. Zhang](https://advanced.onlinelibrary.wiley.com/authored-by/Zhang/Dieqing), *Adv. Funct. Mater.* **2025**, 2425956.
29. L. Ding, M. Li, Y. Zhao, H. Zhang, J. Shang, J. Zhong, H. Sheng, C. Chen, [J. Zhao](https://www.sciencedirect.com/author/7410312341/jincai-zhao), *Appl. Catal. B Environ.* **2020**, *266*, 118634.
30. H. Belhadj, A. Hakki, P. Robertson, D. Bahnemann***,*** [*Phys. Chem. Chem. Phys*.](https://doi.org/10.1039/1463-9084/1999) **2015**, *17*, 22940.
31. X. Dong, W. Cui, H. Wang, J. Li, Y. Sun, H. Wang, Y. Zhang, H. Huang, F. Dong, *Sci. Bull*. **2019**, *64*, 669.
